# Neuronal CDK5RAP3 deficiency leads to encephalo-dysplasia *via* upregulation of N-glycosylases and glycogen deposition

**DOI:** 10.1038/s41420-025-02414-y

**Published:** 2025-04-06

**Authors:** Fanghui Chen, Minghui Xiang, Zhipeng Wang, Fan Yang, Junzhi Zhou, Zihan Deng, Susu Wang, Ping Li, Jieqi Tew, Wei Zhang, Honglin Li, Yong Teng, Xiaobin Zhu, Yafei Cai

**Affiliations:** 1 College of Animal Science and Technology, Nanjing Agricultural University, Nanjing, 210095 China; 2 Department of Human Anatomy, Bengbu Medical College, Bengbu, 233030 China; 3 School of Basic Medicine, Guangdong Medical University, Dongguan, 523808 China; 4 Department of Biochemistry and Molecular Biology, Medical College of Georgia, Augusta University, Augusta, GA 30912 USA; 5 Department of Hematology and Medical Oncology, Winship Cancer Institute, Emory University School of Medicine, Atlanta, GA 30322 USA; 6 Department of Spine Surgery and Musculoskeletal Tumor, Zhongnan Hospital of Wuhan University, Wuhan City, 430071 China

**Keywords:** Gene regulation, Morphogenesis

## Abstract

CDK5RAP3 is a binding protein of CDK5 activating proteins and also one of the key co-factors of the E3 enzyme in the UFMylation system. Several reports have implicated the involvement of CDK5 and other components of the UFMylation system in neuronal development and multiple psychiatric disorders. However, the precise role of CDK5RAP3 in neurons remains elusive. In this study, we generated CDK5RAP3 neuron-specific knockout mice (CDK5RAP^F/F^: Nestin-Cre). CDK5RAP3 conditional knockout (CDK5RAP3 CKO) mice exhibited severe encephalo-dysplasia and a slower developmental trajectory compared to wild-type (WT) mice and succumbed to postnatal demise by day 14. Transcriptome sequencing unveiled that CDK5RAP3 deficiency affects synapse formation, transmembrane trafficking and physiological programs in the brain. Morphological analysis demonstrated that neuronal CDK5RAP3 deficiency leads to increased SLC17A6 and N-glycosylase (RPN1 and ALG2) protein expression, and while causing endoplasmic reticulum (ER) stress. In vitro experiments utilizing CDK5RAP3^F/F^: ROSA26-ERT2Cre MEFs were conducted to elucidate similar mechanism following CDK5RAP3 deletion. Both in vivo and in vitro, CDK5RAP3 deficiency significantly increased the expression of N-glycosylases (RPN1 and ALG2), as well as the total amount of glycoproteins. CDK5RAP3 may potentially maintain a balance by enhancing the degradation of RPN1 and ALG2 through proteolytic degradation pathways and autophagy. This study underscores the indispensable role of CDK5RAP3 in neuronal development and sheds new light on drug discovery endeavors targeting early brain abnormalities.

## Introduction

N-glycosylation is among the most ubiquitous post-translational modifications (PTMs), characterized by oligosaccharides covalently linked to asparagine residues within the polypeptide chain and is highly conserved [1]. This protein modification occurs from the rough endoplasmic reticulum to the Golgi apparatus and is critical for protein folding and stability. It plays a pivotal role in cellular secretion, immunity, cytoskeletal composition, proliferation, and apoptosis, among other functions [1]. During N-glycosylation, RPN1 identifies nascent peptide chains on the ER and attaches mannose to the asparagine residue of the peptide chain [2]; ALG2 is an enzyme that further modifies the glycan after glycan-peptide linkage formation and translocation from the ER [1]. During neural development, N-glycosylated proteins are instrumental in nerve fiber formation and neurotransmitter secretion mediated by synaptic vesicles, thus any disruption in N-glycosylation can profoundly impact organisms [3, 4]. Notably, in mice with neuro-inflammation, there was a significant decrease in the abundance of N-glycans [5]. Aberrant N-glycosylation modifications, whether excessive or deficient, can influence the differentiation and neural development of early neurons and are closely associated with the onset of various brain diseases [6]. N-glycosylation deficiency represents one form of congenital glycosylation disorders (CDGs), characterized by maturation disorders, poor suckling, motor nerve dysfunctions, and developmental delays in affected infants after birth [7]. Moreover, congenital disorders of N-glycosylation can inflict severe damage on the central nervous system of infants, leading to symptoms such as cerebellar atrophy and epilepsy [8]. The loss of N-glycosylated proteins associated with neurons has been implicated in the pathogenesis of Alzheimer’s disease [9, 10]. Although the correlation between different N-glycosylated proteins in the cerebral cortex and the pathogenesis of Alzheimer’s disease varies, there is a significant increase in the number of N-glycosylation sites on proteins in Alzheimer’s pathology [8, 11, 12]. Furthermore, individuals with Alzheimer’s disease exhibit higher glycan levels in the frontal cortex region compared to those without the disease [13]. However, the mechanisms through which excessive N-glycosylation impacts the nervous system remain unclear.

CDK5RAP3 was initially identified as a protein associating with P35, and the activator of the Cyclin-dependent kinase 5 (CDK5) [14, 15]. While CDK5 is widely distributed in organisms, including brain tissue, P35 expression is restricted to the neuron. Early studies reported that CDK5 expression in nerves was closely associated with nerve cell proliferation, differentiation, and synaptic growth [16–21]. Additionally, CDK5 was implicated in synaptic growth [18, 22, 23]. It is well-established that excessive accumulation of P25 and the cleavage product of P35 could lead to hyperphosphorylation of tau protein, exacerbating Alzheimer’s disease with neurodegeneration and disruption of the neuroskeleton [23–28]. Furthermore, CDK5RAP3 was identified as an important component of the UFMylation pathway, and dysregulation of UFMylation can contribute to the onset of neurological disorders [29–33]. The UFMylation system plays a neuroprotective role in the aging process of fruit flies [30], and neuro-deficiency UFMylation can result in microcephaly and neuroinflammation in mice [29, 32, 33], underscoring the critical role of CDK53RAP3 expression in brain neurons for neural development and function. However, despite its role as a binding protein of P35 and a key component of the UFMylation system, studies investigating the impact of dysregulated CDK5RAP3 expression on brain development and function are completely unclear.

In this study, we elucidated for the first time the role of CDK5RAP3 in postnatal brain development and function in mice, which was achieved by constructing conditional knockout (CDK5RAP3 CKO) mice with CDK5RAP3-deficient neurons. We systematically monitored their postnatal behavior and growth development, while also exploring the functional changes within neurons in mouse brain with or without neuronal CDK5RAP3 deficiency.

## Results

### CDK5RAP3 expression is elevated in the hippocampus, cerebral cortex and choroid plexus in mouse brain

**Fig. 1:**
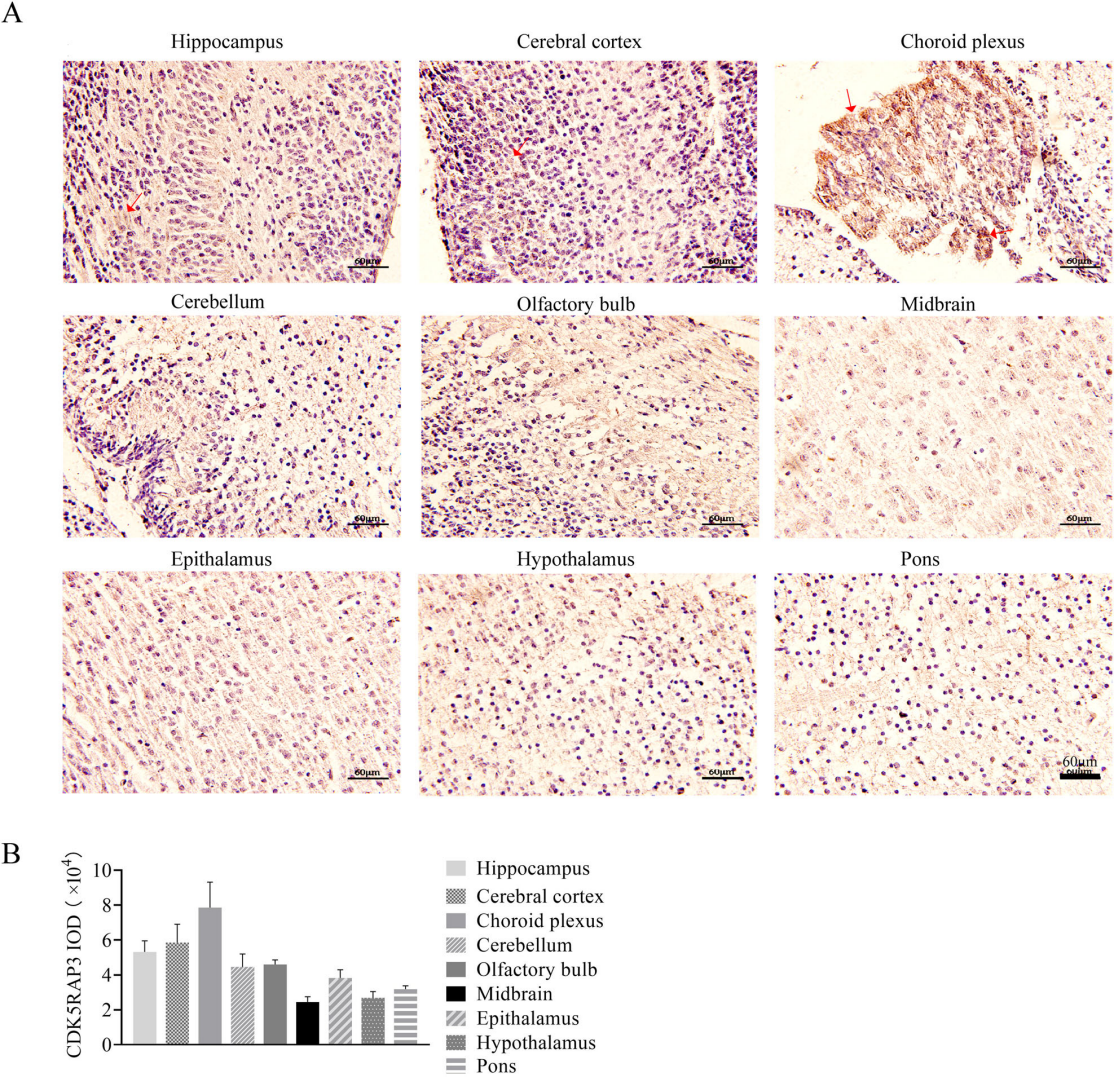
CDK5RAP3 is significantly highly expressed in hippocampus, cerebral cortex and choroid plexus. **A** IHC staining of CDK5RAP3 on mouse hippocampus, cerebral cortex, choroid plexus, cerebellum, olfactory bulb, midbrain, epithalamus, hypothalamus and pons. **B** Integrated optical density in mouse brain regions of (**A**) (*n* = 3 mice/group).

To investigate the expression of CDK5RAP3 in various regions of mouse brain, immunohistochemical (IHC) staining was conducted on brain tissues from wild-type (WT) / C57Bl6J mice (Fig. 1A). The examined brain regions included the hippocampus, cerebral cortex, choroid plexus, cerebellum, olfactory bulb, midbrain, epithalamus, hypothalamus, and pons. Our findings revealed that CDK5RAP3 was consistently expressed across the above brain tissues both in the cytoplasm and nucleus of cells (Fig. 1B), with expression regions of top 3 were the hippocampus, cerebral cortex and choroid plexus compared to other regions examined in this study (Fig. 1B). Meanwhile the representative image of negative control staining (without primary antibody) in the brain tissue was shown no positive staining (Fig. S7). These results suggest that CDK5RAP3 expression may play a prominent role in these three brain tissues.

### Neuron-specific loss of CDK5RAP3 induces pre weaning mortality in mice

**Fig. 2:**
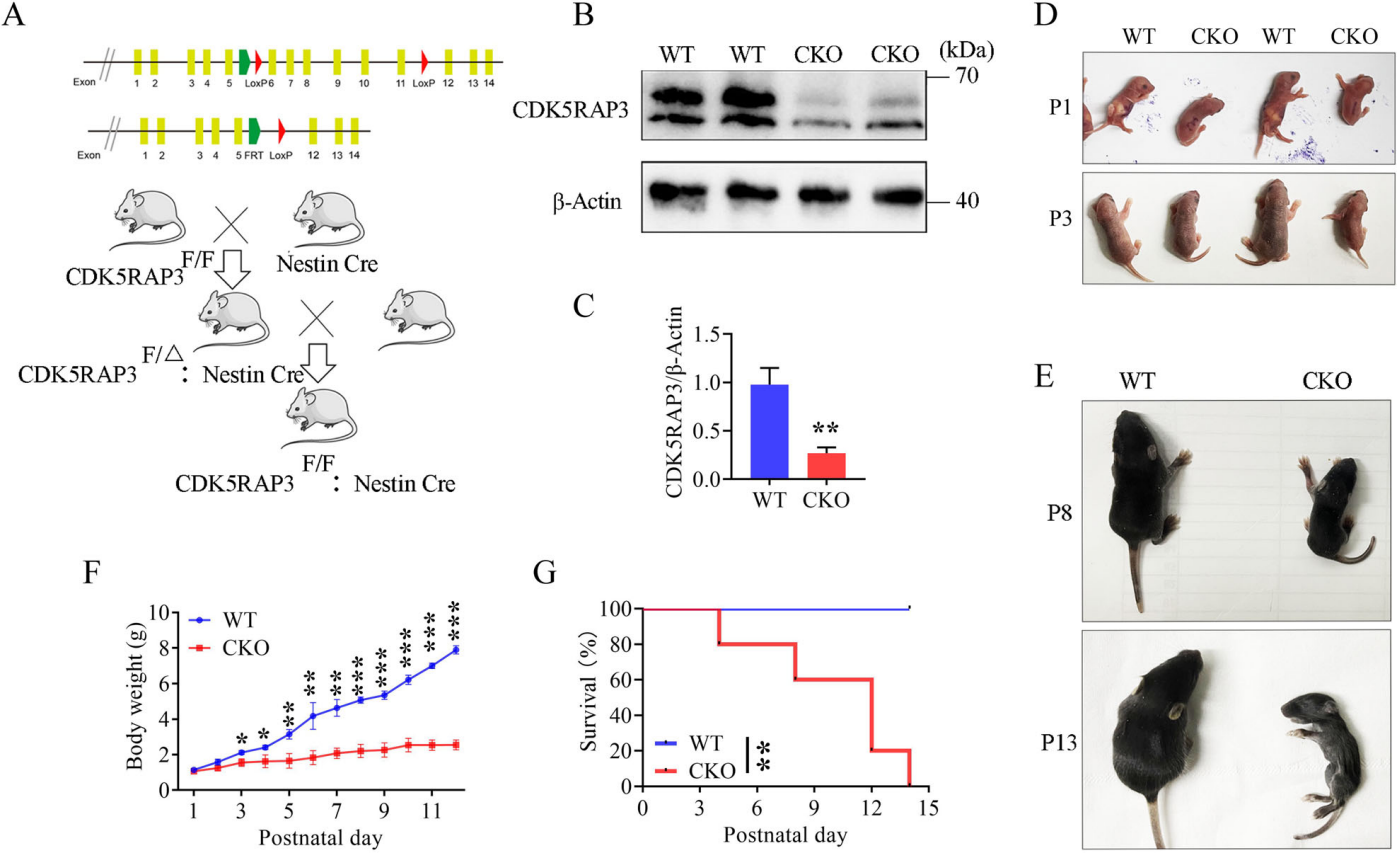
Neuron-specific depletion of CDK5RAP3 causes pre weaning mortality in mice. **A** Generation of neuron-specific CDK5RAP3 conditional knockout (CKO) mice. The CDK5RAP3^F/F^: Nestin-Cre genotype is CKO mice, while other genotypes are collectively referred to as WT mice. **B** Western blot analysis of CDK5RAP3 in WT and CKO mice brains (*n* = 3 mice/group). **C** Statistics on protein levels of CDK5RAP3. The level was normalized to β-Actin (*n* = 3 mice/group). **D** Representative pictures of WT and CKO mice at postnatal 1 day (P1) and postnatal 3 day (P3). **E** Representative pictures of WT and CKO mice at postnatal 8 (P8) and postnatal 13 (P13) day. **F** Daily body weight curve of WT and CKO (*n* = 3 mice/group) mice from postnatal 1 day (P1) to postnatal 12 day (P12). **G** Survival curve of WT (*n* = 14 mice/group) and CKO (*n* = 5 mice/group) mice from P1 to P14. Survival curves were compared using the Log-rank (Mantel-Cox) for two groups(*p* = 0.0021). Statistical significance was determined by unpaired, two-tailed Student’s t test. *p < 0.05; **p < 0.01.

To explore the role of CDK5RAP3 in the nervous system, we engineered neuron-specific knockout mice by crossing CDK5RAP3-floxed transgenic mice with Nestin-Cre transgenic mice. (Figs. 2A and S1). Subsequently, CDK5RAP3 conditional knockout mice (CDK5RAP3^F/F^: Nestin-Cre, CKO) were obtained, and knockout efficiency was assessed by WB of the whole brain tissue. The total protein from the whole brain tissue leads to the weaker bands were observed in the neuron-specific CDK5RAP3 knockout mice compared to WT (Fig. 2B, C). Although no significant differences were observed in the body size and weight of CDK5RAP3 CKO mice compared to WT mice at 2 days after birth, the disparity in body size and weight between CDK5RAP3 CKO and WT mice became more pronounced from postnatal day 3 to day 13 (Fig. 2D–F). Additionally, Neuron-specific CDK5RAP3 knockout mice exhibited poor sucking behavior, movement disorders, and gradual mortality within 14 days after birth (Fig. 2G, Fig. Video). These results support the notion that neuronal CDK5RAP3 deficiency in mice leads to dysplasia, movement disorders, and mortality prior to weaning.

### Neuronal CDK5RAP3 deficiency leads to abnormal brain structure and massive neuronal death

**Fig. 3:**
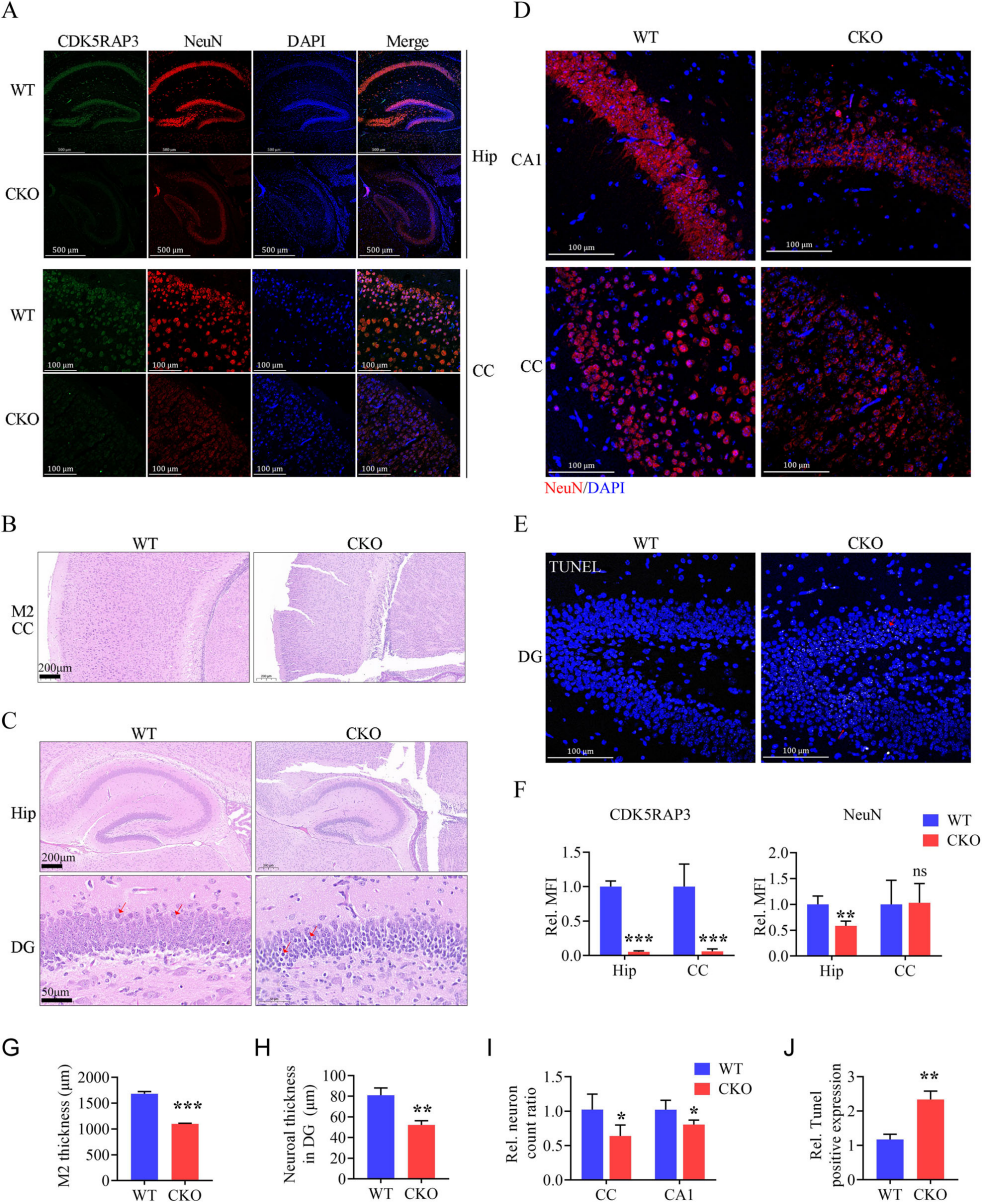
Neuronal CDK5RAP3 deficiency induces abnormal brain structure and massive neuronal death. **A** Co-immunofluorescence staining of CDK5RAP3 (green) and NeuN (red) of hippocampus (Hip) and cerebralcortex(CC) in WT and CKO mice (*n* = 3 mice/group). While the intensity quantitative analysis was show in the (**F**). **B**, **C** Representative images HE staining pictures of cerebral cortex, Hip and dentate gyrus(DG) in the CKO mice compared with WT mice. **D** NeuN(red) staining of CA1 and CCin the CKO mice compared with WT mice. **E** TUNEL staining and (**J**) quantification of positive nuclei analysis in DG (*n* = 3 mice/group). **G** M2 Thickness of cerebral and cortex from CKO and WT mice. **H** Neuroal thickness of cell layer of dentate gyrus pyramidal neuron from CKO and WT mice. **I** Statistic normalized neuron count ratio of CC and CA1 in WT and CKO mice brain (*n* = 3 mice/group). Statistical significance was determined byunpaired, two-tailed Student’s *t* test. ***p < 0.001; **p < 0.01; *p < 0.05.

To comprehensively understand why CDK5RAP3 deficiency in the neuronal system leads to mouse sucking difficulties, growth and movement disorders in mice, and ultimately to death during lactation, immunofluorescence staining, H&E staining, and TUNEL staining were conducted to assess the differences in brain tissue development between WT and CDK5RAP3 CKO mice at postnatal day 1 (P1). The markedly lower levels of CDK5RAP3 expression in the hippocampus and cerebral cortex region of neuron-specific CDK5RAP3 knockout mice compared to WT. Meanwhile, the NeuN expression was notably reduced in the dentate gyrus of CDK5RAP3 CKO mice, which was used as a neuronal marker protein (Fig. 3A, F). Additionally, cortical brain thickness was significantly diminished in mice lacking CDK5RAP3 (Fig. 3B, G), and the hippocampus exhibited reduced size compared to WT mice at P13 (Fig. 3C). Furthermore, the stratification of cells in the dentate gyrus of the hippocampus in the neuron-specific CDK5RAP3 knockout mice were displayed a thinner pyramidal neuron cell layer (Fig. 3C, H) and significant nuclear pyknosis. We concurrently stained and counted neurons in the brains of CDK5RAP3 CKO and WT mice using DAPI and NeuN staining, and found a significant reduction for the number of neurons in the cerebral cortex and CA1 regions of the CKO mouse brain compared to WT mice (Fig. 3D, I). At the same time, significantly more TUNEL-positive cells were detected in the cortical and dentate gyrus regions of neuron-specific CDK5RAP3 knockout mouse brains than WT mice (Fig. 3E, J). While representative genes associated with cell death were validated by RT-qPCR, the mRNA levels of partial genes were increased significantly in the brain tissues from neuron-specific CDK5RAP3 knockout mouse (Fig. S5C). Taken together, our findings strongly suggest that CDK5RAP3 deficiency results in structural abnormalities in the brain and widespread neuronal demise. These collectively represent phenotypes of encephalo-dysplasia.

### Neuronal CDK5RAP3 deficiency affects transport of glutamate neurotransmitters

**Fig. 4:**
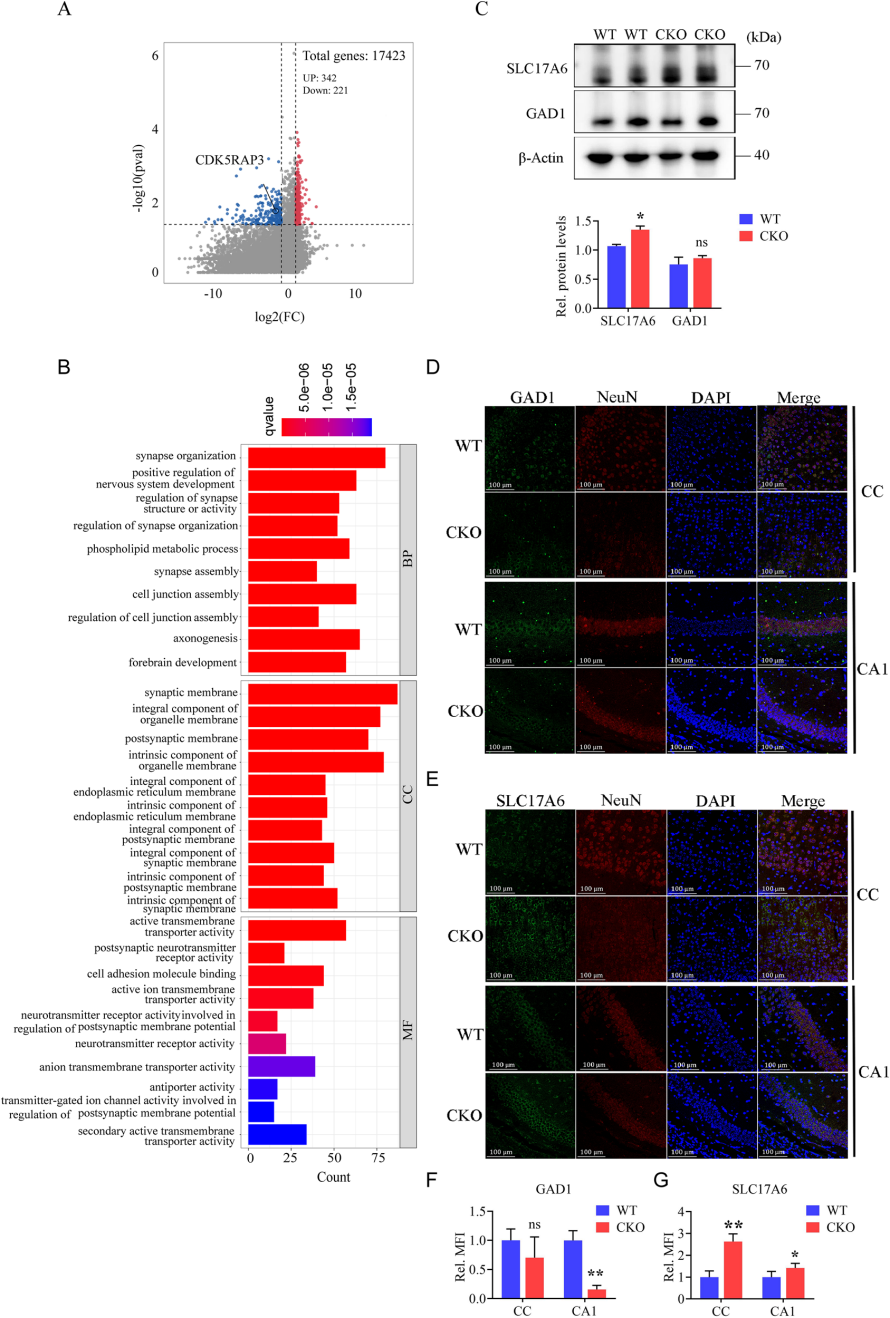
Neuronal CDK5RAP3 deficiency affects transport of glutamate neurotransmitters. **A** Volcano map of transcriptome analysis in WT and CKO mice. **B** GO enrichment analysis of differentiated genes (DEGs). **C** Western blot analysis of SLC17A6 and GAD1 from brain samples extracted from WT and CKO mice (*n* = 3 mice/group). Quantitative data are shown in the below panel. Co-staining with (**D**) GAD1 and NeuN, **E** SLC17A6 and NeuN of CC and CA1. **F**, **G** Quantitative data of the relative fluorescence intensity are shown in the below panel (*n* = 3 mice/group). Statistical significance was determined by unpaired, two-tailed Student’s *t* test. ** *p* < 0.01; * *p* < 0.05.

To investigate the main functional genes affected in the brain of neuron-specific CDK5RAP3 knockout mice, mRNA-seq was performed on brain tissues collected from WT and CDK5RAP3 CKO mice at postnatal day 1 (P1). A total of 563 differentially expressed genes (DEGs) were identified in CDK5RAP3 CKO mice compared to WT mice, with 342 DEGs up-regulated and 221 DEGs downregulated (log2 fold change ≥ |±1.5 | , Fig. 4A). Gene Ontology (GO) analysis revealed significant changes in processes related to “synapses,” “development of the nervous system,” “regulation of synapses,” and “various activities of transmembrane transfer” in CDK5RAP3 CKO mice (Fig. 4B), suggesting that the loss of CDK5RAP3 in neurons primarily leads to synaptic dysfunction.

Disruption in synaptic function and transmembrane activity in CDK5RAP3 CKO mice brain were shown in the GO enrichment analysis, raising the possibility that neurotransmitter generation and transport at the synapse might be affected. We then chose L-glutamatergic and GABAergic neurons, which constitute a significant portion of brain neurons to investigate the synthesis and trafficking of their respective neurotransmitters. In RNA-seq data, although we did not observe significant differences in the expression of marker genes overall associated with the glutamate and GABA (Fig. S2A), so immunofluorescence staining of GAD1 (glutamate decarboxylase 1) and SLC17A6 (solute carrier family 17 member 6) in conjunction with NeuN were conducted to analyze these processes (Fig. 4D, E). Our results displayed a significant down-regulation of GAD1 levels in the CA1 region of the CDK5RAP3 CKO mice, whereas there was no significant decrease of GAD1 levels in the cerebral cortex of CDK5RAP3 CKO mice compared with WT mice at postnatal day 1 (Fig. 4F). Additionally, SLC17A6 levels were apparently up-regulated in both the cerebral cortex and CA1 regions of CDK5RAP3 CKO mice (Fig. 4E, G). As expected, the WB results showed an obviously increased expression of SLC17A6 protein in the brain tissue of CDK5RAP3 CKO mice at postnatal day 1. But no major differences in GAD1 protein levels between two groups (Fig. 4C). The findings suggest that neuronal CDK5RAP3 deficiency results in elevated expression of SLC17A6, thereby disrupting L-glutamate transport. This disruption in L-glutamate transport represents one of the contributing factors to motor disorder observed in CDK5RAP3 CKO mice.

### Neuronal CDK5RAP3 deficiency induces endoplasmic reticulum stress and N-glycosylase deposition in mouse brain tissues

**Fig. 5:**
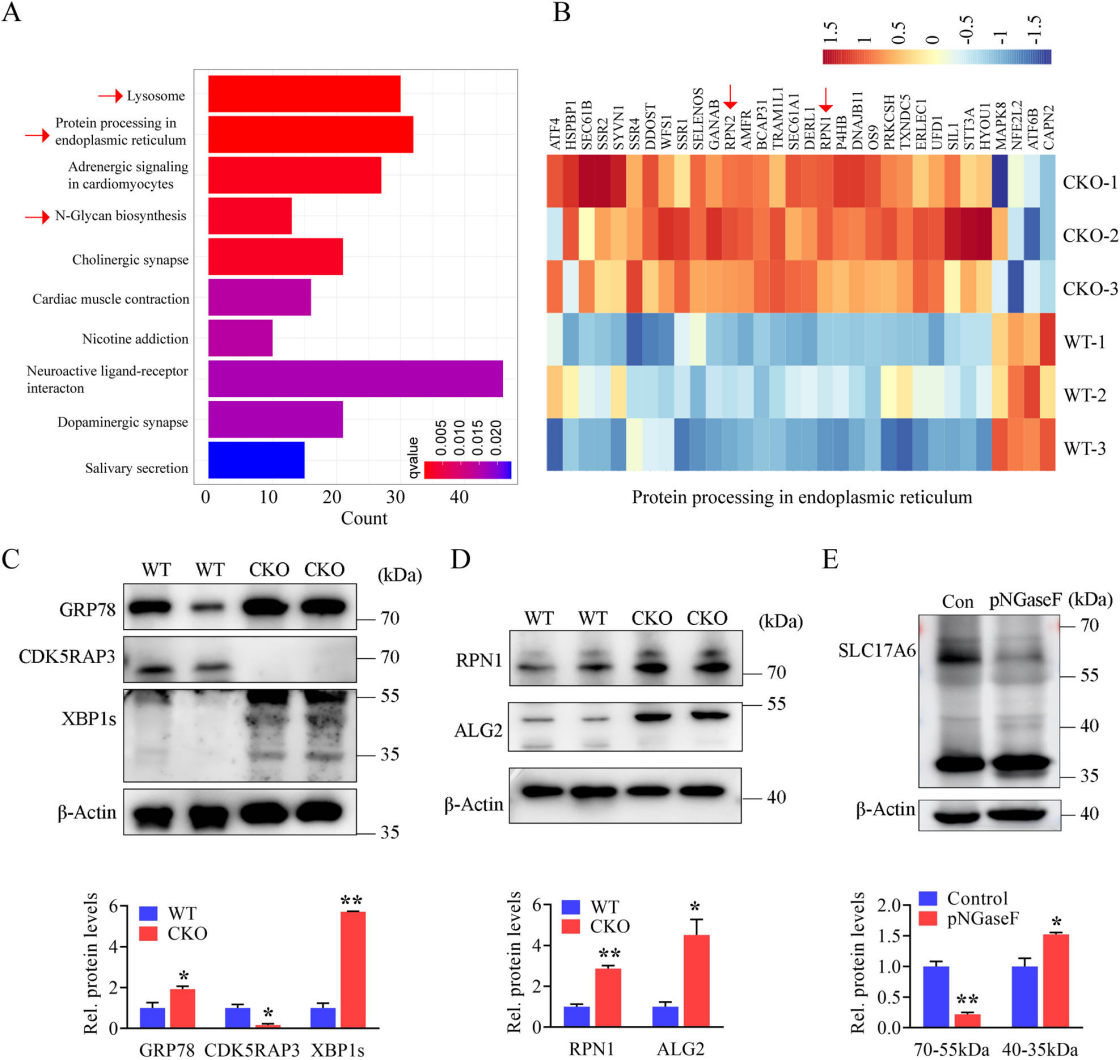
Neuronal CDK5RAP3 deficiency causes endoplasmic reticulum (ER) stress and N glycosylase deposition. **A** KEGG enrichment analysis of DEGs. **B** Heatmap of protein process in ER related DEGs. **C** Western blot analysis of GRP78, CDK5RAP3, XBP1s and (**D**) RPN1 and ALG2 protein levels in brain tissues extracted from WT and CKO mice 1 day postpartum. Quantitative data are shown in the lower panels (*n* = 3 mice/group). **E** Western blot analysis of SLC17A6 protein levels before and after N-glycosylation removal. Quantitative data are shown in the lower panels (*n* = 3 mice/group). Con: Control, proteins extracted from adult mouse brain without any treatment; pNGaseF: proteins extracted from adult mouse brain and incubated with PNGaseF for 8 h at 37 °C. Statistical significance was determined by unpaired, two-tailed Student’s t test. * *p* < 0.05; **p < 0.01.

Intriguingly, Kyoto Encyclopedia of Genes and Genomes (KEGG) analysis unveiled dysregulation in several pathways, notably ‘lysosome’, ‘protein processing in the endoplasmic reticulum (ER)’, ‘adrenergic signaling in cardiomyocytes’, and ‘N-Glycan biosynthesis’, in CDK5RAP3 CKO mice (Fig. 5A). Heatmap was shown differentially expressed genes associated with protein processing in ER (Fig. 5B). Consistently, the levels of ER stress-related proteins GRP78 and XBP1s were significantly upregulated in the brain of CDK5RAP3 CKO mice via WB analysis (Fig. 5C). Moreover, three obvious upregulated genes (RPN1, RPN2, and DDOST) involved in N-glycan biosynthesis were observed (Fig. 5B). Consequently, the protein levels of RPN1 (localized on ER) and ALG2 (not localized on ER), two key regulators in N-glycan biosynthesis, were significantly increased in the brains of CDK5RAP3 CKO mice compared with WT mice (Fig. 5D).

Due to the observed increased SLC17A6 protein expression of CDK5RAP3 CKO mice in Figs. 4 and S2A, then considering SLC17A6’s role as a membrane transporter for glutamate, we hypothesize its potential glycosylation. Thus, further validation is warranted to confirm whether it undergoes N-glycosylation modification. N-glycosylation was removed from brain tissue of WT mice, and it was observed that the SLC17A6 protein band originally modified by N-glycosylation appears at 65 kDa. However, upon deglycosylation, the band decreases to 40-35 kDa, with minimal change observed in total expression levels (Fig. 5E). This suggests that SLC17A6 expression can be influenced by N-glycosylase.

### Neuronal CDK5RAP3 deficiency induces ER stress, cell death and N-glycosylase abnormalities in embryonic fibroblast cells (MEFs)

**Fig. 6:**
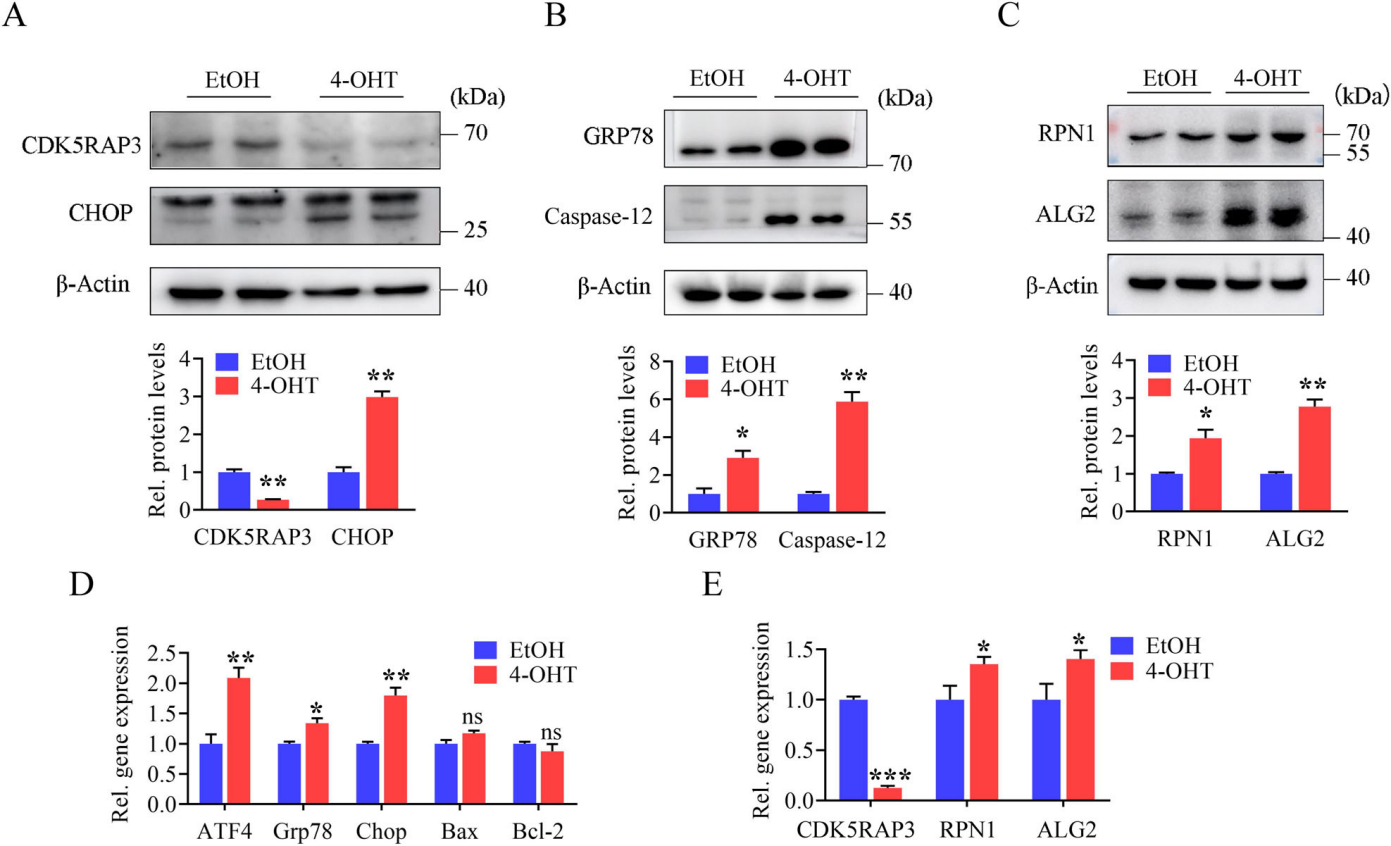
CDK5RAP3 depletion results in ER stress, cell death and N-glycosylase abnormalities in MEFs. **A**, **B** Western blot analysis of the key markers involved in ER stress, cell death after knockdown of CDK5RAP3 in MEFs compared with control. Quantitative data are shown in the lower panel. **C** Western blot analysis of ALG2 and RPN1 in MEFs beforeand after knocking down CDK5RAP3, and statistical results of protein expression were shown in the lower panel. All western blots were independently repeated at least three times with consistent results. **D**, **E** Q-PCR determination of mRNA levels of ATF4, GRP78, Chop, Bax, Bcl-12 RPN1 and ALG2 in MEFs before and after knocking down CDK5RAP3. All western blots and Q-PCR were independently repeated in triplicates. Statistical significance was determined by unpaired, two-tailed Student’s t test.***p < 0.001; **p < 0.01; *p < 0.05.

To gain deeper insights into why CDK5RAP3 deficiency leads to abnormal N-glycosylation, MEFs derived from mice with CDK5RAP3^F/F^: ROSA26-ERT2Cre were selected. CDK5RAP3 gene knockout was induced in these MEFs using Tamoxifen (4-OHT) to assess the occurrence of abnormal N-glycosylation. In CDK5RAP3 knockdown MEFs, the levels of GRP78, Chop and Caspase-12 proteins were significantly elevated by WB analysis (Fig. 6A, B), and the mRNA expressions of ATF4, GRP78, and CHOP were also upregulated (Fig. 6D). The genes expression trend of ER stress markers was coincident with that detected by WB. Consistently, similar to observations in the brain of CDK5RAP3 CKO mice (Fig. S5A), the protein levels and mRNA expressions of N-glycosylases (RPN1 and ALG2) were notably increased in CDK5RAP3 knockdown MEFs (Fig. 6C, E). These findings confirm that CDK5RAP3 deletion induces ER stress and increased levels of N-glycosylases in MEFs.

To ascertain whether ER stress leads to the upregulation of N-glycosylases, we further treated MEFs with 3 μM thapsigargin (TG) for 8 hours to induce ER stress. The experimental results revealed that under conditions of ER stress, there were no significant changes in the protein expression of CDK5RAP3 and RPN1 in MEFs. However, there was a remarkably significant increase in the protein expression levels of ALG2 in CDK5RAP3 knockout MEFs (Fig. S3). This suggests that the increase in N-glycosylases due to CDK5RAP3 deficiency may be partially induced by ER stress.

### Loss of CDK5RAP3 leads to increase of glycoproteins

**Fig. 7:**
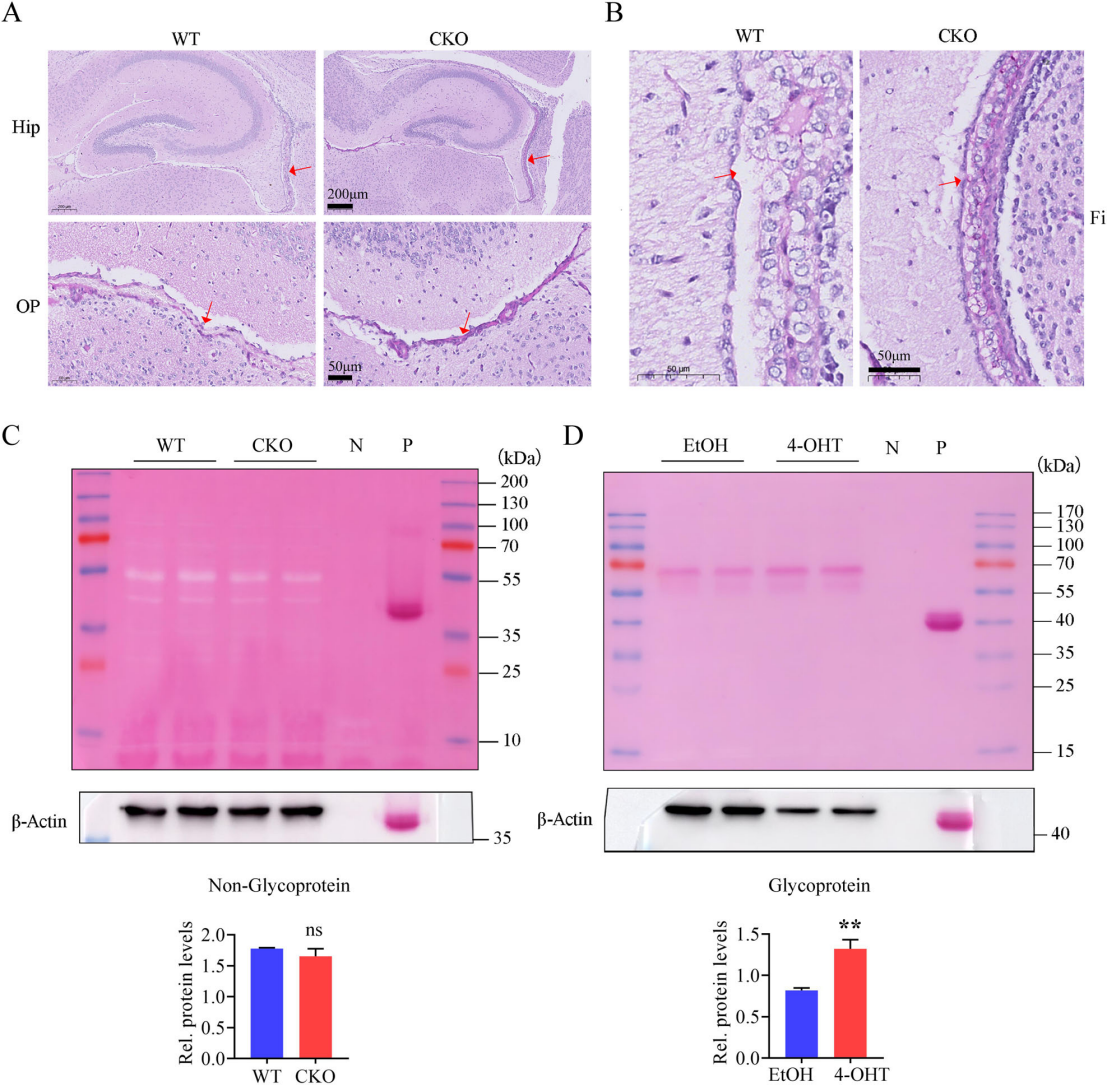
CDK5RAP3 depletion leads to increase of glycoproteins. **A** Representative picutres of PAS staining in the regions of neuronal-CDK5RAP3 knockout mice brain including the Hip (hippocampus), OP (optic nerve layer) and (**B**) fimbria of hippocampus compared with WT mice (*n* = 3 mice/group). **C** Glycoprotein staining of brain tissues from WT and CKO mice (*n* = 3 mice/group). **D** Glycoprotein staining before and after knocking down CDK5RAP3 in MEFs. Statistical analysis of protein expression levelswere displayed in the lower panel. N: Negative control, P: Positive control. Statistical significance was determined by unpaired, two-tailed Student’s t test. **p < 0.01.

Because N-glycosylase was significantly increased in the brains of neuron-specific CDK5RAP3 knockout mice and MEFs upon CDK5RAP3 knocked down, then whether CDK5RAP3 affects the protein levels of N-glycosylated proteins was explored. Notably, clear purple-red staining indicative of glycogen deposition was observed in the optic nerve layer of the superior colliculus and the nerve bundles at the edge of the hippocampal fimbriae in PAS-stained brain slices of CKO mice compared WT mice (Fig. 7A, B). Moreover, while the total amount of non-glycoproteins (the white band on the PVDF membrane) in the brains of CDK5RAP3 deficiency mice was slightly lower than that of WT mice, the difference was not statistically significant (Fig. 7C). Conversely, a significant increase in the total amount of glycoproteins was observed in MEFs with CDK5RAP3 knocked out (Fig. 7D). This finding indicates that the absence of CDK5RAP3 leads to an increase in both glycoproteins and glycogen deposition.

### CDK5RAP3 participates in N-glycosylase protease degradation and autophagy

**Fig. 8:**
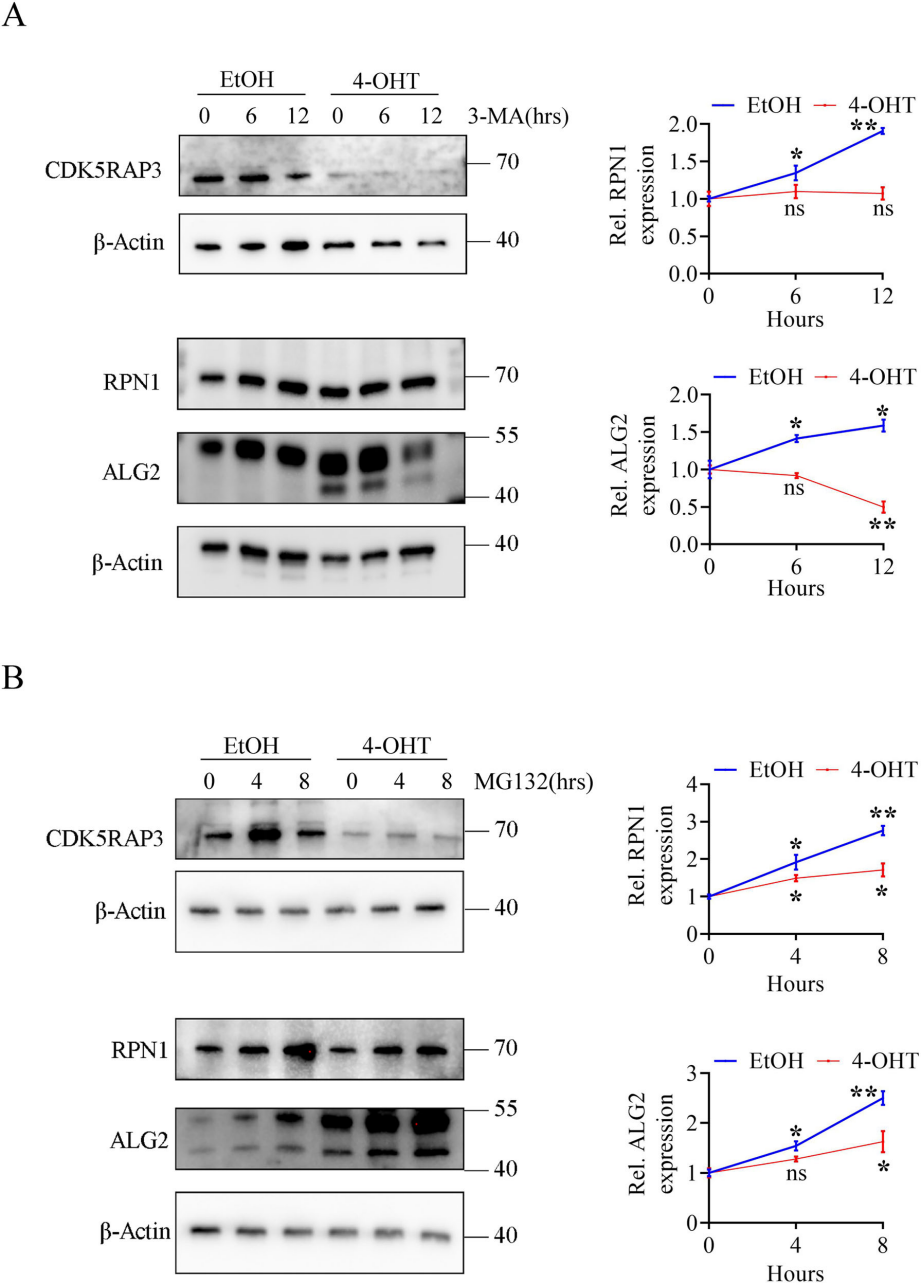
CDK5RAP3 balances the content of N-glycosylase by promoting protease degradation and autophagy. **A** Western blot analysis of RPN1 and ALG2 in MEFs before and after knocking down CDK5RAP3. MEFs were pretreated with 3-MA for indicated times. Statistics results from three independent experiments are shown in the right panel. **B** Western blot analysis of RPN1 and ALG2 in MEFs before and after knocking down CDK5RAP3. MEFs were pretreated with 40 μM cycloheximide (CHX) for 12 h accompanied by 2 μM MG132 treatment for the indicated times. Statistics results from three independent experiments are shown in the right panel. Statistical comparisons were determined by one- way analysis of variance (ANOVA) analysis with Turkey’s multiple comparisons. **p < 0.01.* *p* < 0.05.

Previous research in our laboratory demonstrated that the degradation pathway of autolysosomes was inhibited in breast cells with CDK5RAP3 knockdown [34]. In this study, KEGG enrichment analysis and Heatmap of Lysosome of transcription data shows the significant difference expression between WT and CDK5RAP3 CKO mice brain (Figs. 5A and S2B). MEFs were treated with 3-Methyladenine (3-MA, 5 mM) for different time periods as indicated, which was widely used as an autophagy inhibitor. Interestingly, we observed that the protein levels of RPN1 and ALG2 in CDK5RAP3 knockout MEFs did not upregulate following 3-MA treatment, suggesting that autophagy inhibition does not impact the expression of these proteins under the condition of CDK5RAP3 knockout (Fig. 8A).

Next, CDK5RAP3 knockout MEFs (4-OHT groups) were treated with CHX to inhibit protein synthesis and measured the protein stability over time compared with control (EtOH groups). Surprisingly, RPN1 and ALG2 were less degraded by proteases in 4-OHT groups than EtOH groups, indicating increased stability of RPN1 and ALG2 upon CDK5RAP3 depletion (Fig. 8B). Based on these results, we hypothesized that CDK5RAP3 maintains homeostasis of N-glycosylase protease degradation and autophagy.

## Discussion

**Fig. 9:**
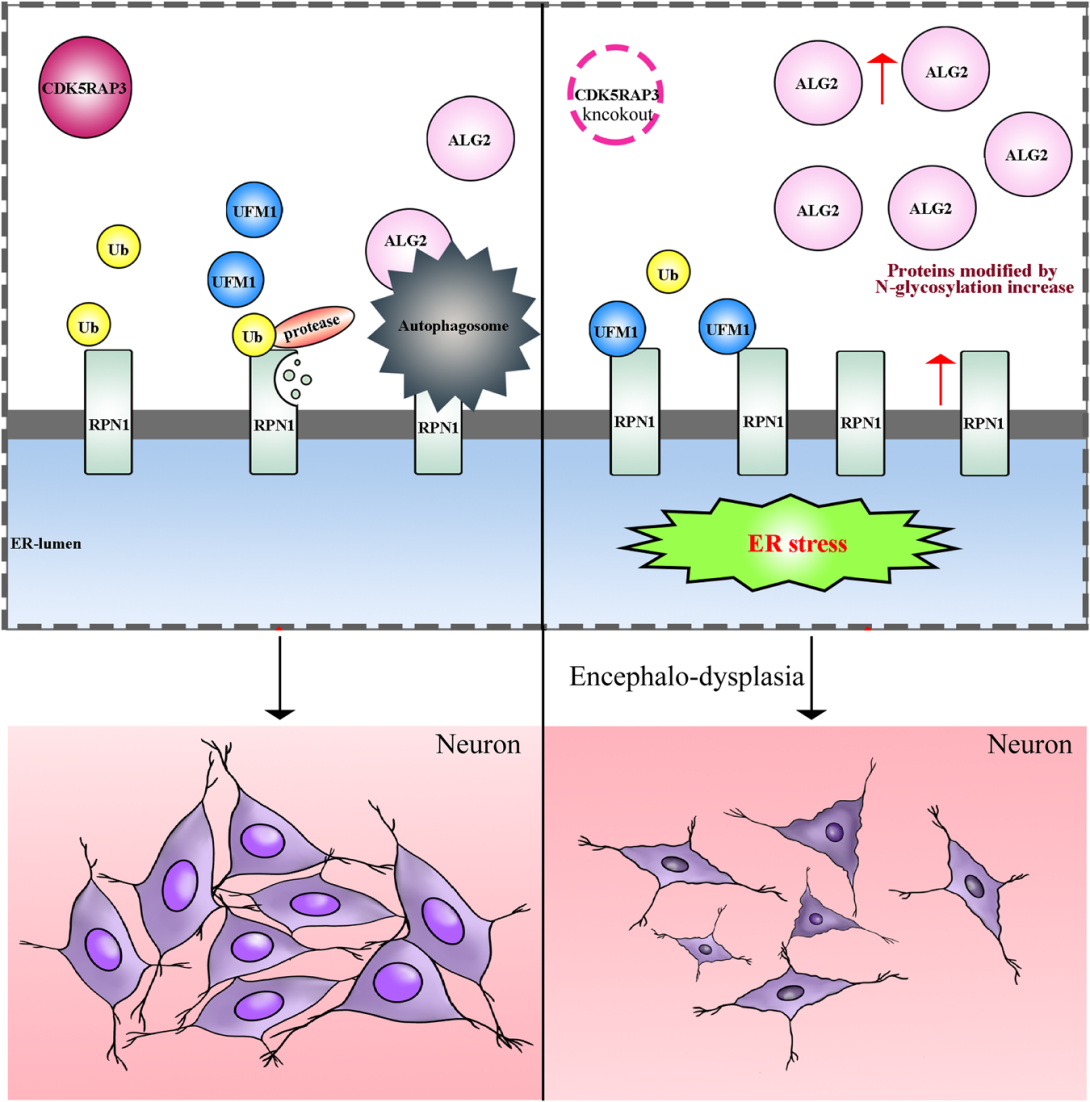
A proposed model illustrating the potential mechanisms of encephalo-dysplasia induced by CDK5RAP3 deficiency during the lactation period of mice. Encephalo-dysplasia and lethality were observed in the CDK5RAP3 neuron specific knockout mice model during the lactation period for the first time. Furthermore, ALG2 and RPN1 protein expression were upregulated significantly induced by the absence of CDK5RAP3, and that upregulation could be associated with N-glycosylase protein degradation, and that degradation is a crucial underlying mechanism associated with neurodevelopmental processes.

Through the establishment of CDK5RAP3 neural knockout mice, we have uncovered novel insights into the biological significance of CDK5RAP3 in neurodevelopment. Our findings elucidate that the absence of CDK5RAP3 in neurons results in encephalo-dysplasia in mice, culminating in lethality during the lactation period. Notably, our study also reveals a pivotal role for CDK5RAP3 in modulating protein N-glycosylation within cells, mediated via regulation of N-glycosylase protein degradation, marking a significant advancement in understanding the molecular mechanisms underlying neurodevelopmental processes (Fig. 9).

CDK5RAP3 serves as a critical interacting factor within the UFMylation system, and its deficiency leads to dysregulation of UFMylation. Prior investigations have demonstrated that defective expression of other components of UFMylation (UBA5, UFC1, UFL1) in neurons results in UFMylation dysregulation, manifesting in microcephaly, seizures, and motor impairments in neonates [29, 32, 33]. Consistently, our experiments revealed phenotypes akin to microcephaly and motor impairments in neuron-specific CDK5RAP3 knockout mice, albeit without the occurrence of seizures. Mice with neuronal loss of UFL1 exhibit pronounced inflammation in the brain [33]. However, in our experiments, transcriptomic analysis of CDK5RAP3 CKO mice did not reveal significant alterations in inflammatory factors (Fig. S2C). Additionally, we confirmed that the absence of CDK5RAP3 in neurons does not impact the mRNA expression of other components of the UFMylation system besides Ufbp1 (Fig. S4A, S4B).

In the framwork of the UFMylation system, a large protein complex consisting of the E3 enzyme UFL1, CDK5RAP3, and UFBP1 was identified, localized within the rough ER, which correlates with ER stress, unfolded protein response (UPR), and ER-associated degradation (ERAD) [35, 36]. UFM1 has been shown to facilitate micro-autophagy within the endoplasmic reticulum (ER) [30, 37–39]. Transcriptomic sequencing of CDK5RAP3 CKO and WT mice, along with mRNA detection in brain tissues, revealed consistent alterations (Fig. S5A, S5B). ER homeostasis is crucial for maintaining normal neuronal development and function in the brain. ER stress often leads to various neurodegenerative diseases, motor impairments, retinal diseases, and significant neuronal loss [40, 41]. Therefore, CDK5RAP3 in neurons may regulate normal brain development by maintaining ER homeostasis, and it may also influence N-glycosylation through its regulation of ER homeostasis.

Further exploration of the phenotype from CDK5RAP3 deficiency, which leads to increased N-glycosylase protein level, revealed a significant elevation of the N-glycosylated protein SLC17A6 in the brains of CDK5RAP3 CKO mice. SLC17A6, also known as VGLUT2 or DNPI, functions similarly to VGLUT1, residing on synaptic vesicles within glutamate neurons and facilitating glutamatergic signaling through neurotransmitter transport via endocytosis and exocytosis [42, 43]. Primarily implicated in conveying excitatory signals linked to perception and autonomous respiration, SLC17A6 exhibits diminished involvement during physical activity [44–47]. Investigation into the VGLUTs family in the mouse brain indicates predominant SLC17A6 expression approximately two weeks before birth, with VGLUT1 and associated proteins increasing thereafter [48, 49]. Despite exhibiting normal embryonic development, mice lacking SLC17A6 expression in neurons perish shortly after birth due to respiratory failure [50]. Abnormal elevation of SLC17A6 has been observed in Parkinson’s disease patients’ brains [51]. We postulate that the heightened SLC17A6 expression in CDK5RAP3 CKO mice intensifies their perception of sensory stimuli, potentially leading to excessive glutamate transmission and subsequent neuronal toxicity and demise. Reduced SLC17A6 expression correlates with heightened activity in SOD1 (G93A) neurons in mice [52]. Additionally, SLC17A6 interacts with CDK5, promoting CDK5/P25-mediated inflammatory pain in inflammation-induced thermal hyperalgesia [47].

CDK5RAP3 has been reported to interact with the CDK5 activating protein [14, 15], suggesting its potential influence on mouse brain nerve development through CDK5-associated pathways, such as SLC17A6/CDK5/P25 [47]. Previous studies have highlighted the roles of CDK5 and its activator P25 in neuron proliferation, differentiation, and synaptic growth [17, 18, 20, 21, 28]. Consistently, Gene Ontology (GO) analysis of transcriptome data from brain tissues of neuron-specific CDK5RAP3 knockout mice revealed enrichment in pathways linked to synapse function (Fig. 5A). Thus, CDK5RAP3 likely modulates the expression of genes involved in synapse-related pathways by influencing CDK5 activity.

Given that several N-glycosylases involved in the biological process of N-glycosylation are situated on the ER (such as RPN1, RPN2, and DDOST), it is plausible that CDK5RAP3 may modulate the expression of N-glycosylases via the UFMylation system. Previous studies suggested that RPN1 shares similarities with RPL26, with ubiquitin and UFM1 competing for binding sites on the protein [53]. CDK5RAP3 has been identified as a critical component of the protein complex composed of E3 ligases in the UFMylation system, with specific knockout of CDK5RAP3 in the liver leading to alterations in UFM1-bound substrates [54].

Our experiments further demonstrated that within 12 hours of CHX treatment, the changes of RPN1 protein levels were relatively small in CDK5RAP3 knockout MEFs compared with control despite prolonged treatment with MG132 (Fig. 8B). Additionally, western blot analysis revealed alterations in UFM1 protein bands in CDK5RAP3-deleted MEFs (Fig. S6B). Furthermore, Ubiquitin (Ub) protein from the brains of CDK5RAP3 CKO mice was also displayed changes via western blot (Fig. S6C), and using CO-IP assays, we found that CDK5RAP3 could not interact with RPN1 in the normal brain tissue from WT mice (Fig. S6A). These findings suggest that the presence of CDK5RAP3 may inhibit the replacement of Ubiquitin by UFM1 on RPN1, thereby impeding RPN1 ubiquitination.

Congenital Glycosylation Deficiency Disorders (CDGs) encompass a spectrum of disorders, wherein defective expression of N-glycosylases serves as one of the causative factors [55]. Patients with CDGs typically exhibit postnatal growth retardation, abnormal motor regulation in the nervous system, immunodeficiency, gastrointestinal dysfunction, and other organ dysfunctions [56–58]. In this study, we confirmed that the loss of CDK5RAP3 in neurons and MEFs leads to increased protein levels of N-glycosylases (RPN1 and ALG2) within the cells. Therefore, CDK5RAP3 could represent a potential therapeutic target for treating CDGs resulting from deficiency in N-glycosylases.

In summary, the absence of CDK5RAP3 in neurons leads to postnatal encephalo-dysplasia and lethality during the lactation period in mice. This phenotype may share similarities with brain developmental defects resulting from dysregulated UFMylation due to deficiencies in the expression of E1, E2, and E3 enzymes involved in UFMylation. However, the phenotype is not entirely consistent. Additionally, the loss of CDK5RAP3 may also potentially disrupt the function of CDK5 in neurons, leading to increased expression of SLC17A6, aberrant glutamate neurotransmitter transport, and subsequently affecting neuronal activity. The absence of CDK5RAP3 in cells also leads to an increase in neuronal death by inducing ER stress and upregulating N-glycosylases. However, the molecular mechanism of CDK5RAP3 was involved in the protein levels of N-glycosylases through ER stress, UFMylation, or autophagy remains incompletely understood and requires further study This study confirms the indispensable role of CDK5RAP3 in postnatal brain development in animals, thereby offering new therapeutic targets for early brain disorders.

## Materials and methods

### Animals, cell lines and reagents

To generate CDK5RAP3 neuron specific knockout mice model, CDK5RAP3 Floxed mice and Nestin-Cre mice were mated to generate homozygous mice, the following primers were used for genotyping of CDK5RAP3^F/F^ Nestin-Cre mice: CDK5RAP3-1 (TAGCTCGGGGCTCAGACG CTCTGA), CDK5RAP3-2 (TTATCTGCTCTTCCCGCTAGAATA); CDK5RAP3^F/F^ PCR condition: 94 °C/4 min, 92 °C/45 s, 55 °C/45 s, 72 °C/45 s, 40 cycles, 72 °C/10 min; WT (−/−): a lower band, Hetero (+/−): upper and lower bands, Homo (+/+): upper band. Nestin-Cre-1 (TTGCTA AAGCGCTACATA GGA), Nestin-Cre-2 (GCCTTATTGTGGAAGGACTG), Nestin-Cre-3 (CCTTCCTGAAGCAGT AGAGCA), Nestin-Cre PCR condition: 94 °C/4 min, 92 °C/45 s, 68 °C/45 s, 72 °C/45 s, 35 cycles, 72 °C /10 min; Nestin-Cre (-): band size about 250 bp, Nestin-Cre (+): band size about 150 bp (Fig. S1).

CDK5RAP3 knockout MEFs were isolated E13 day mouse embryos (genotyping CDK5RAP3^F/F^: ROSA26-Cre/ERT2), washed by PBS, bladed by sterile razor until it becomes possible to pipette. The tissues were digested with trypsin and collagenase. Finally, the MEFs were transfected with lentivirus and screened using puromycin to collect the positive immortalized cells. MEFs were treated 4-OHT (2 μM, Sigma) for 5 days to induce CDK5RAP3 knockdown, the control group was added the equal amount of ethanol (EtOH). All the cells were ensured no mycoplasma contamination via using Mycoplasma Detection Kit (Lonza).

MEFs were treated with 5 mM 3-MA (MCE, #HY-19312) for 6 hours and 12 hours to inhibit the autophagy. MEFs were pretreated with 40 μM CHX (MCE, #HY-12320) for 12 h to inhibit protein synthesis, then incubated with 2 μM MG132 (Sigma, #M7449) for the indicated times to observe the stability of target protein. MEFs were pretreated 3 μM TG (MCE, #HY-13433) for 8 hours to induce ER stress.

### Western blot

The entire brain of the mouse or cells were collected and lysed with appropriate amount of complete RIPA lysis buffer (Thermo, RIPA buffer, #89901) including protease inhibitors cocktail (Invitrogen, #P1005). Measure the concentration of proteins using BCA assay kit (Beyotime, #P0010, Shanghai, China). Then protein was analyzed on denatured SDS-PAGE finally.

Primary antibodies used in this study included β-Actin (#AC004); CDK5RAP3 (#A13128); SLC17A6 (#A15177); GAD1 (#A2938); GRP78 (#A11568); XBP1s (#A17007); RPN1 (#A12497); ALG2 (#A7843); Chop (#A20987); Caspase-12 (#A0217); UFM1 (#A15843); Ubiquitin (#A19686). Secondary antibodies included goat anti-rabbit IgG (#AS014) and goat anti-mouse IgG (#AS003). The above antibodies were purchased from ABclonal Technology (Wuhan, China).

CDK5RAP3 (Abcam, #ab157203, Cambridge, UK); NeuN (Abcam, #ab104224, Cambridge, UK); Fluorophore-conjugated secondary antibodies, anti-mouse IgG (Abcam, #ab150117, Cambridge, UK) and anti-rabbit IgG (Abcam, #ab150075, Cambridge, UK).

### Immunohistochemistry and immunofluorescence staining

Polyvinylidene fluoride membrane protein PAS staining: After transferring the western blotting test protein onto the polyvinylidene fluoride membrane, use the PAS staining kit (Real Times, #RTD6501, Beijing, China) to process and stain the membrane according to the instructions.

For immunohistochemistry, tissue sections were dewaxed and rehydrated before antigen thermal repair treatment, add 0.5% Triton X-100 to cover the tissue for 1 h at 37 °C, and primary antibodies were incubated at 4 °C overnight. DAB (Vector, #SK4105, Burlingame, USA) staining was performed and ready to observe. For immunofluorescence, the tissue was covered with fluorescent secondary antibodies, then slides were sealed with antifade mounting medium with DAPI under dark conditions. Observed and collected the photos under a laser scanning confocal microscope (Carl Zeiss, Zeiss LSM 900, Jena, Germany).

### RT-qPCR

The tissues and cells were subjected to RNA extraction using the AFTSpin Tissue/Cell Fast RNA Extraction Kit for Animal (ABclonal, #RK30120, Wuhan, China), and the experimental steps were strictly carried out according to the instructions. After measuring the RNA concentration, reverse transcription was performed using HiScript II Q Select RT SuperMix for qPCR (Vazyme, Nanjing, China). qRT-PCR was performed using SYBR Green premix (Vazyme, Nanjing, China) on the Step-One PCR system (Applied Biosystems 7500, CA, USA). The primers used for qRT-PCR are listed in Supplementary Table 1, and all expression data were presented using β- Actin used as a control for standardization.

### Transcriptome sequencing and data analysis

RNA-seq was performed by (Guangzhou Deaou, China). Differential analysis was conducted using the GENE DENOVO Audi Cloud platform; GO and KEGG enrichment analysis were obtained using R language software using ‘clusterProfiler’, enrich plot ‘,’ ggplot ‘,’ ggnewscale ‘,’ DOSE ‘,’ stringer ‘, and’ org. Bt. eg. db ‘ R packages. DEGs refer to genes with log2 fold change ≥ |±1.5| and an adjusted p-value (FDR) < 0.05.

### Protein N-glycosylation

The protein concentration of the brain tissue was adjusted to 1 mg/ml; Performed N-glycosylation treatment on the protein using PNGase F Deglycosylation Kit (Beyotime, #P2318S, Shanghai, China), strictly following the instructions. Afterwards, the protein was denatured using 5X SDS-PAGE Sample Loading Buffer and validated by western blotting.

### Co-immunoprecipitation

Protein extraction was performed using immunoprecipitation assay kit (Proteintech, #PK10008, Wuhan, China), and the primary antibody required for detection was added. Added cleaned agarose beads after overnight rotation at 4 °C, following the instructions for incubation, elution, and denaturation. The final samples were subjected to western blotting to detect the target protein level.

### Data statistics and analysis

Perform statistical analysis and plot charts using GraphPad Prism v.9.0 software. Datra were expressed as mean ± standard deviation (SD). Comparison of two groups was performed using unpaired Student’s t-test. Comparison of more than two groups, One-way analysis of variance (ANOVA) followed by Tukey’s multiple testing correction was used in the study. Log-rank (Mantel-Cox) survival analysis was used to determine the impact of CDK5RAP3 expression on overall survival of mice. *P* values less than 0.05 were indicated statistically significant.

## Supplementary information

Representative electropherograms of mice genotype in order to obtain neuron-specific CDK5RAP3 knockout mice.

Heatmaps of representative DEGs associated with the Glu & GABA system (A), lysosome (B) and inflammation (C) were screened from RNAseq analysis for WT and CKO mice (n=3 mice/group).

WB analysis of CDK5RAP3, p-IRE1α, GRP78, RPN1 and ALG2 proteins in the MEFs that were treated with 3μM Thapsigargin (TG) for 8 hours (n=3).

Heatmaps of representative DEGs associated with the Ufmylation system (A), and the genes also were measured by RT-qPCR (n=3 mice/group).

RT-qPCR analysis of representative genes associated with UPR system (A), ERAD (B) and Cell death (C) (n=3 mice/group).

Co-immunoprecipitation (co-IP) of CDK5RAP3 and RPN1 in the normal brain tissue from WT mice (A). Western blot analysis of Ufm1 (B) and Ubiquitin (C) levels in MEFs (n=3).

Representative image of negative control staining in the brain tissue (without primary antibody).

Supplementary Figure Legends

RT-qPCR primer sequences.

CDK5RAP3 CKO mice exhibited poor sucking behavior, movement disorders, and gradual mortality within 14 days after birth

Original Western blot Figures

## Data Availability

All datasets are available from the corresponding author upon reasonable request.
