# Supplementary figures and images for: Neuronal CDK5RAP3 deficiency leads to encephalo-dysplasia via upregulation of N-glycosylases and glycogen deposition
Source: Cell Death Discov. 2025 Apr 6;11:146. doi: 10.1038/s41420-025-02414-y (PMC11972371; doi:10.1038/s41420-025-02414-y)

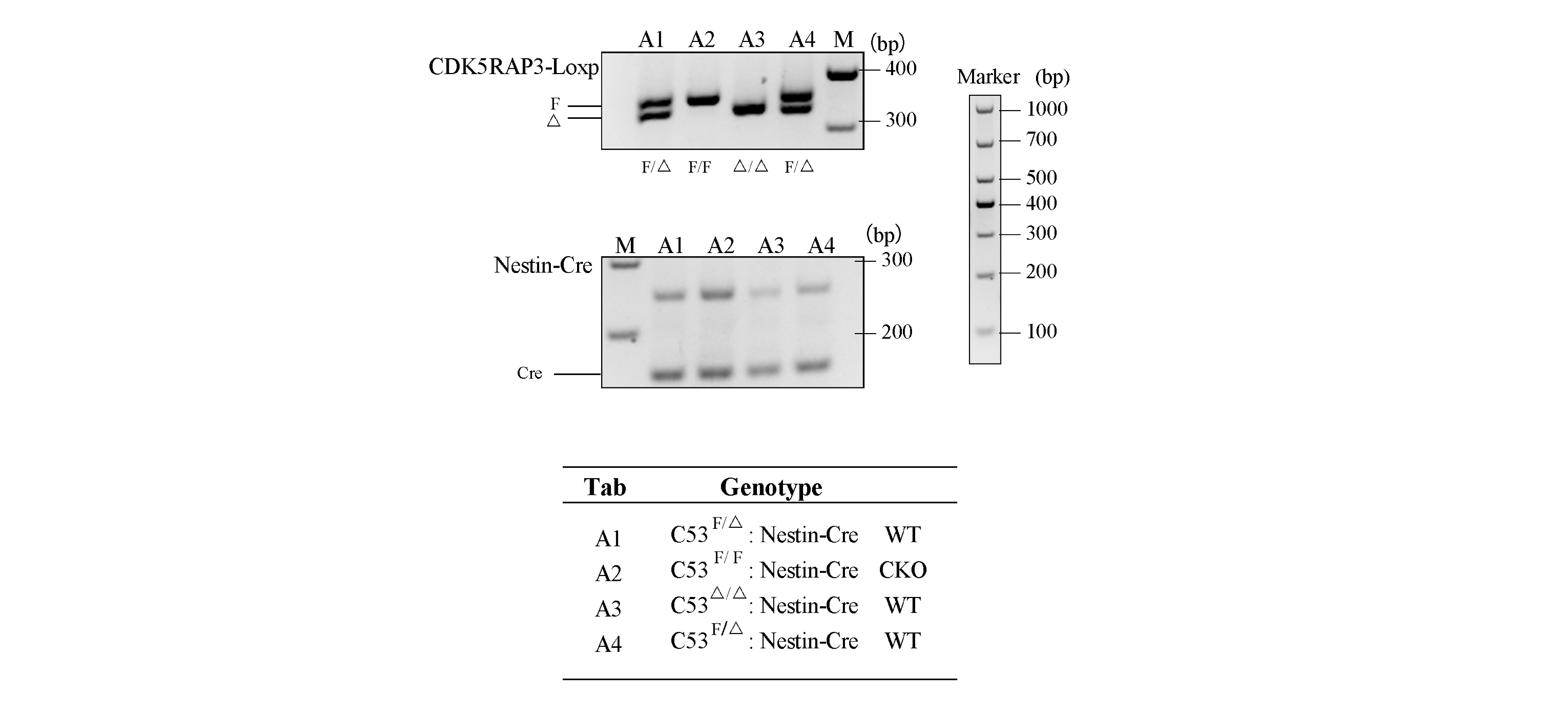

Supplement: Supplementary file 1 — Representative electropherograms of mice genotype in order to obtain neuron-specific CDK5RAP3 knockout mice. [file 41420_2025_2414_MOESM1_ESM.tif]

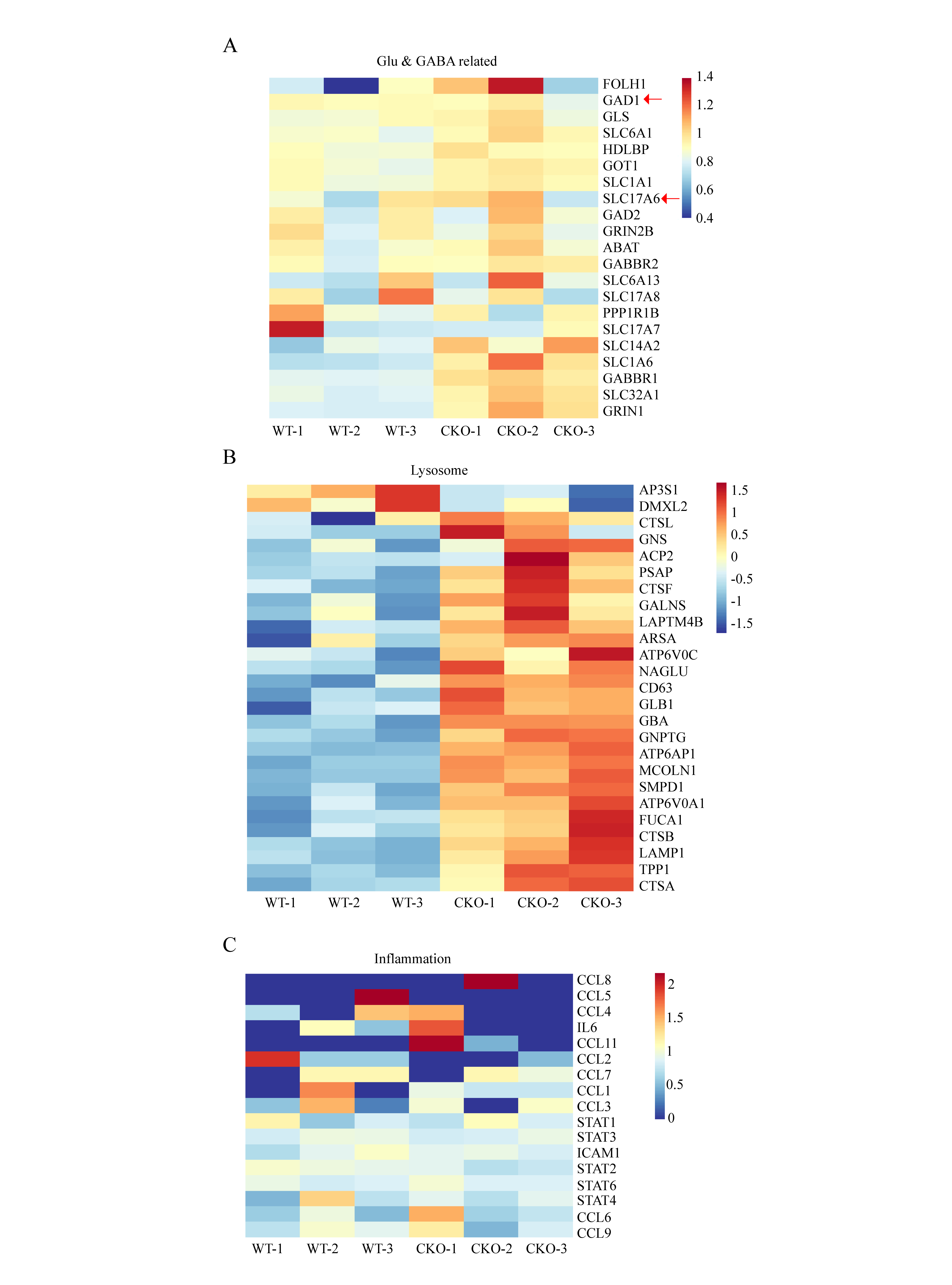

Supplement: Supplementary file 2 — Heatmaps of representative DEGs associated with the Glu & GABA system (A), lysosome (B) and inflammation (C) were screened from RNAseq analysis for WT and CKO mice (n=3 mice/group). [file 41420_2025_2414_MOESM2_ESM.tif]

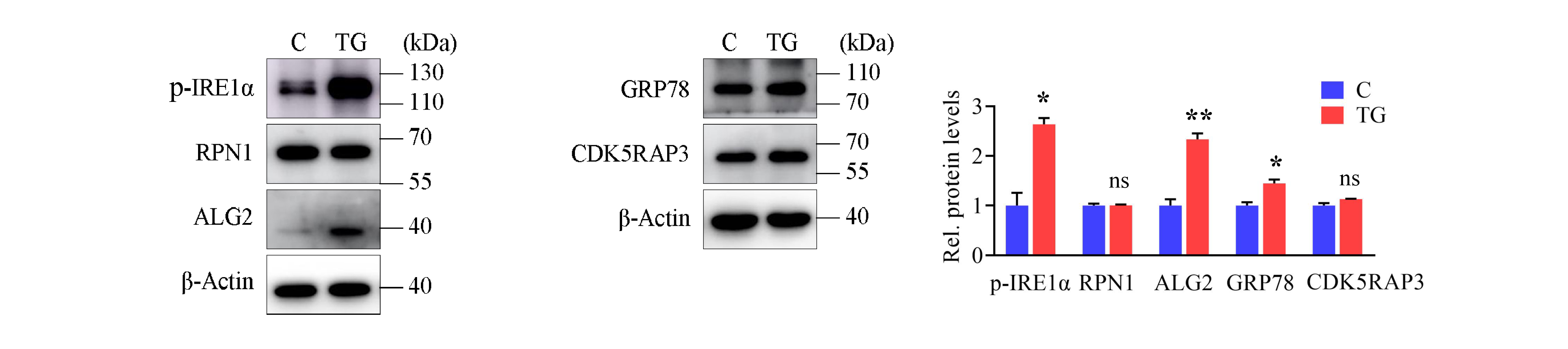

Supplement: Supplementary file 3 — WB analysis of CDK5RAP3, p-IRE1α, GRP78, RPN1 and ALG2 proteins in the MEFs that were treated with 3μM Thapsigargin (TG) for 8 hours (n=3). [file 41420_2025_2414_MOESM3_ESM.tif]

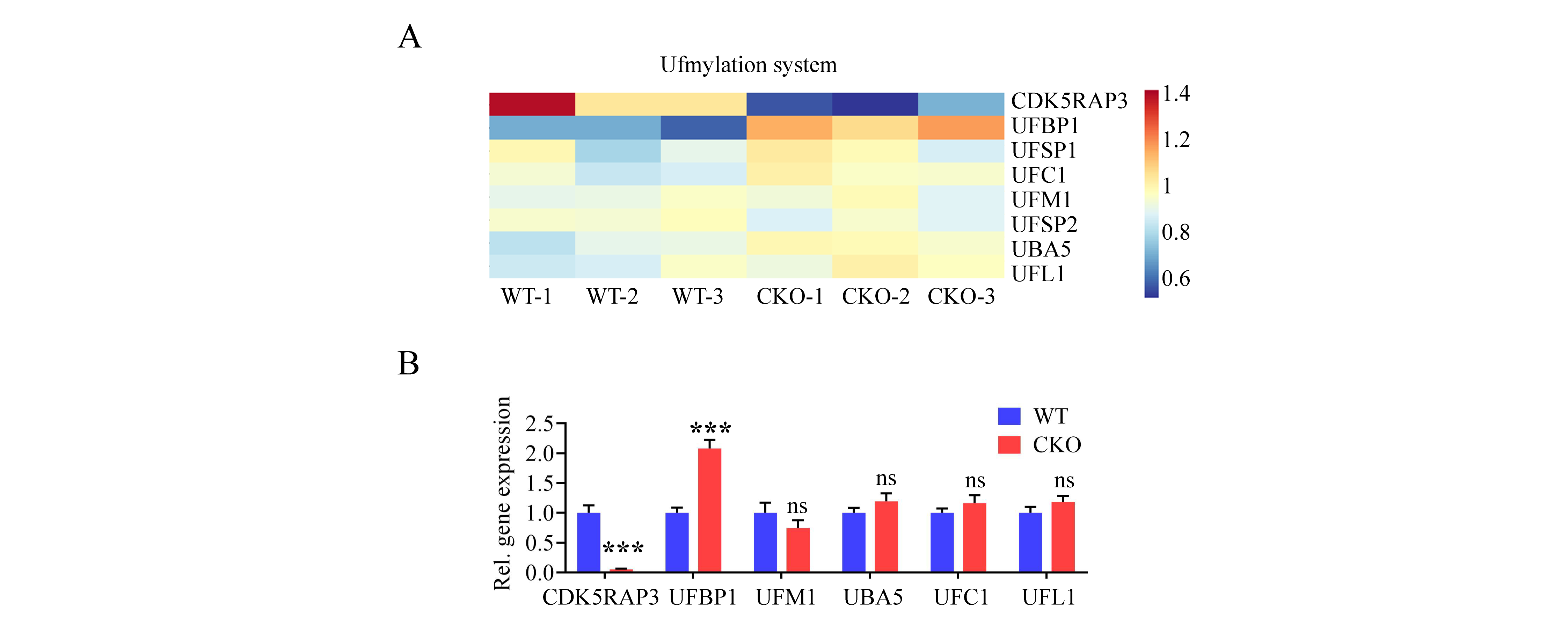

Supplement: Supplementary file 4 — Heatmaps of representative DEGs associated with the Ufmylation system (A), and the genes also were measured by RT-qPCR (n=3 mice/group). [file 41420_2025_2414_MOESM4_ESM.tif]

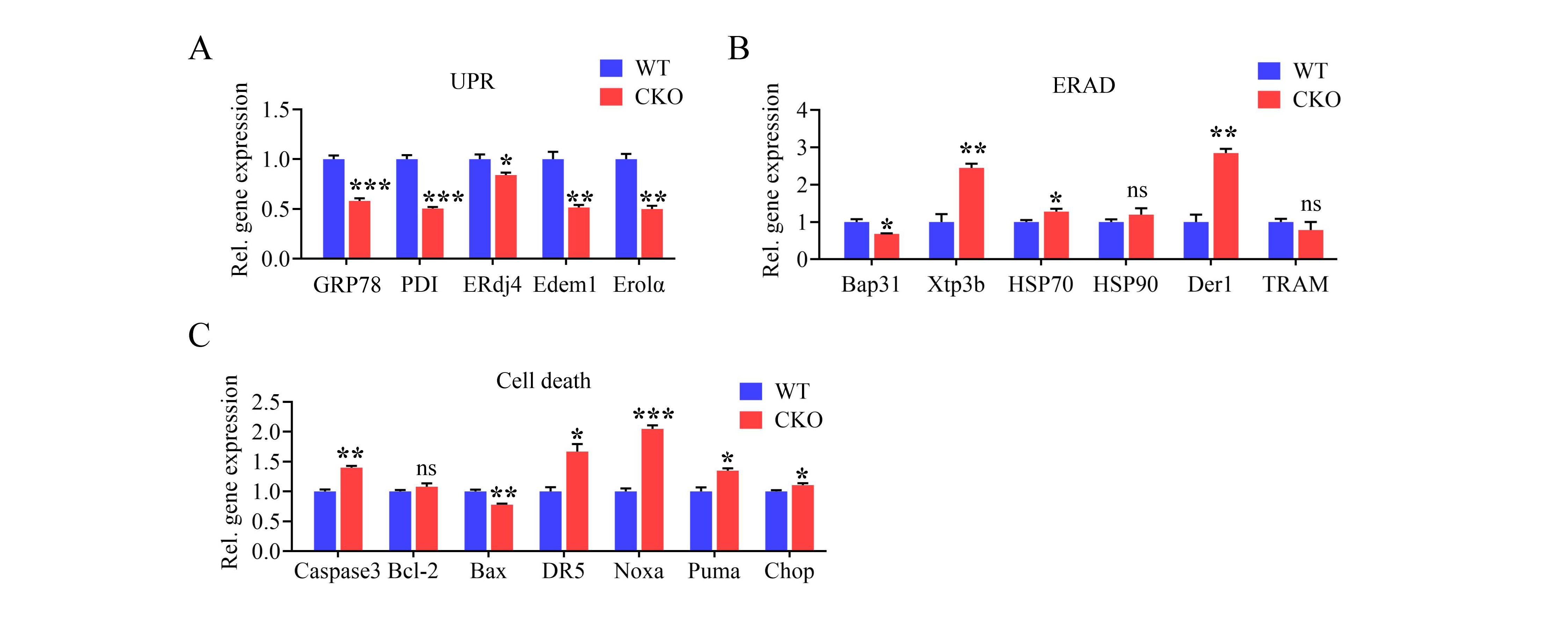

Supplement: Supplementary file 5 — RT-qPCR analysis of representative genes associated with UPR system (A), ERAD (B) and Cell death (C) (n=3 mice/group). [file 41420_2025_2414_MOESM5_ESM.tif]

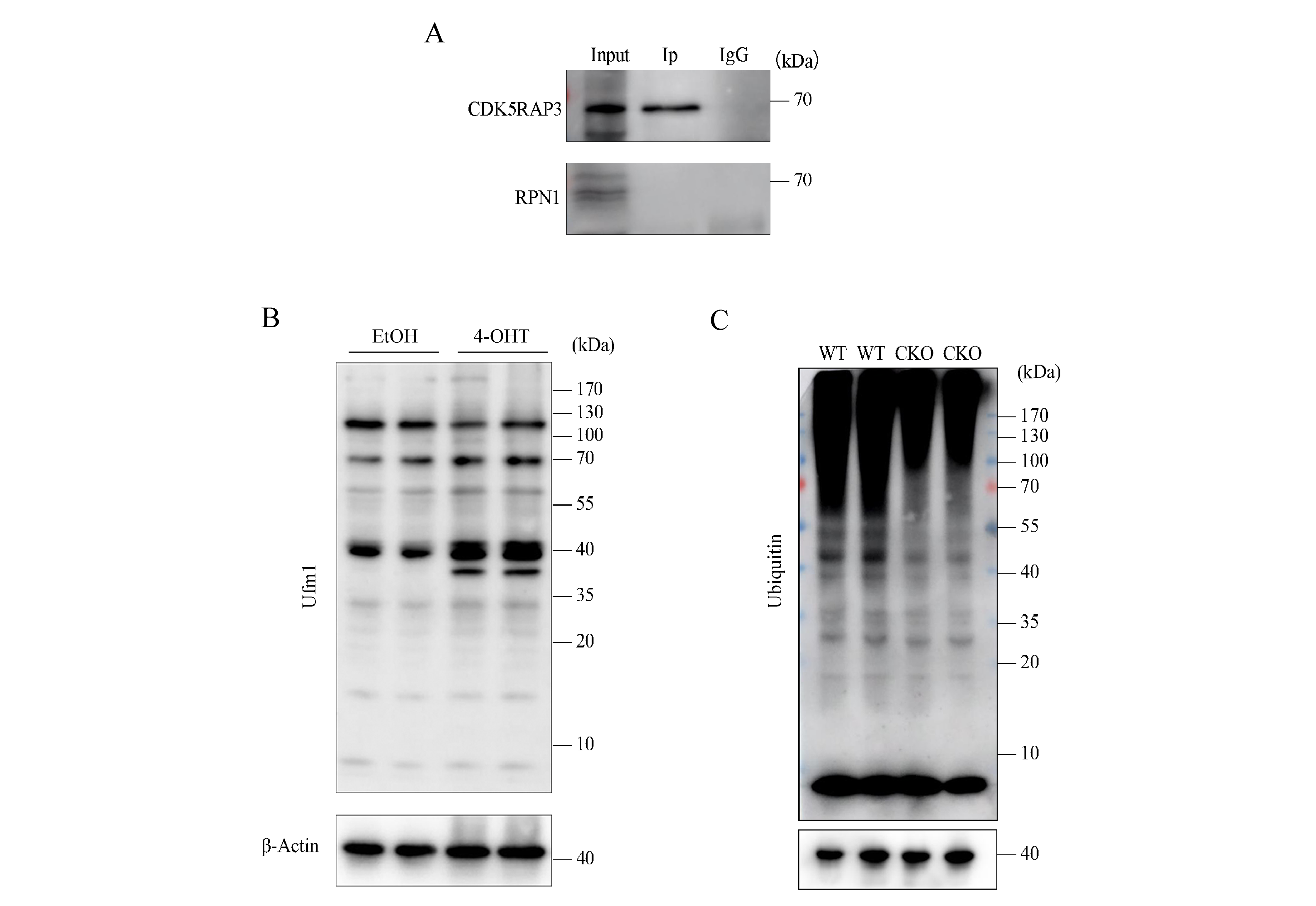

Supplement: Supplementary file 6 — Co-immunoprecipitation (co-IP) of CDK5RAP3 and RPN1 in the normal brain tissue from WT mice (A). Western blot analysis of Ufm1 (B) and Ubiquitin (C) levels in MEFs (n=3). [file 41420_2025_2414_MOESM6_ESM.tif]

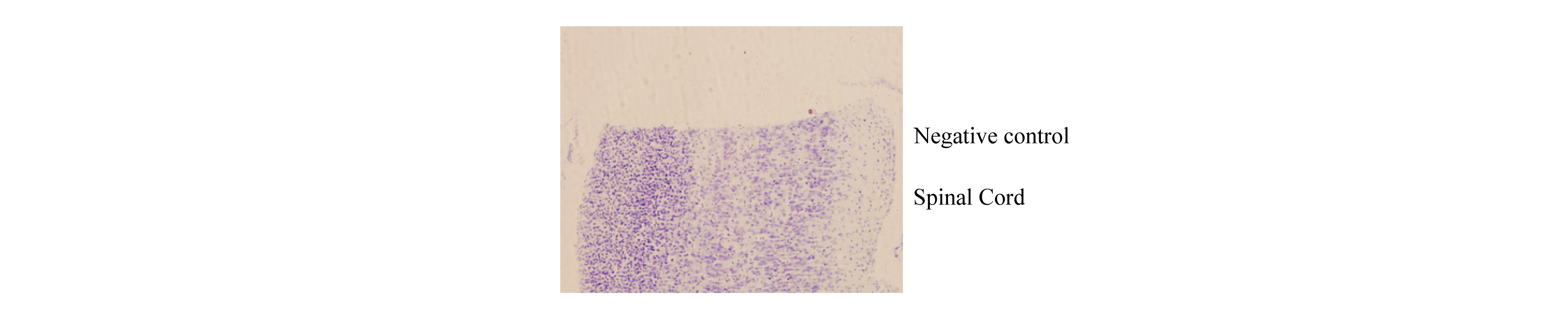

Supplement: Supplementary file 7 — Representative image of negative control staining in the brain tissue (without primary antibody). [file 41420_2025_2414_MOESM7_ESM.tif]
