# Supplementary material for: Neuronal CDK5RAP3 deficiency leads to encephalo-dysplasia via upregulation of N-glycosylases and glycogen deposition
Source: Cell Death Discov. 2025 Apr 6;11:146. doi: 10.1038/s41420-025-02414-y (PMC11972371; doi:10.1038/s41420-025-02414-y)
Supplement: Supplementary file 8 — Supplementary Figure Legends [file 41420_2025_2414_MOESM8_ESM.docx]

**Supplementary Figure Legends**

**Fig. S1 Representative electropherograms of mice genotype in order to obtain neuron-specific**

**CDK5RAP3 knockout mice.**

**Fig. S2 Heatmaps of representative DEGs associated with the Glu & GABA system (A), lysosome**

**(B) and inflammation (C) were screened from RNAseq analysis for WT and CKO mice (n=3**

**mice/group).**

**Fig. S3 WB analysis of CDK5RAP3, p-IRE1α, GRP78, RPN1 and ALG2 proteins in the MEFs**

**that were treated with 3μM Thapsigargin (TG) for 8 hours (n=3). Statistical significance was**

**determined by unpaired, two-tailed Student’s t test. **p* < 0.05, ***p* < 0.01.**

**Fig. S4 Heatmaps of representative DEGs associated with the Ufmylation system (A), and the genes also were measured by RT-qPCR (n=3 mice/group). Statistical significance was** **determined by unpaired, two-tailed Student’s t test. ***p* < 0.01.**

**Fig. S5 RT-qPCR analysis of representative genes associated with UPR system (A), ERAD (B) and**

**Cell death (C) (n=3 mice/group). Statistical significance was determined by unpaired, two-tailed**

**Student’s t test. **p* < 0.05, ***p* < 0.01.**

**Fig. S6 Co-immunoprecipitation (co-IP) of CDK5RAP3 and RPN1 in the normal brain tissue from WT mice (A). Western blot analysis of Ufm1 (B) and Ubiquitin (C) levels in MEFs (n=3).**

**Fig. S7 Representative image of negative control staining in the brain tissue (without primary antibody).**
