# Supplementary material for: Neuronal CDK5RAP3 deficiency leads to encephalo-dysplasia via upregulation of N-glycosylases and glycogen deposition
Source: Cell Death Discov. 2025 Apr 6;11:146. doi: 10.1038/s41420-025-02414-y (PMC11972371; doi:10.1038/s41420-025-02414-y)
Supplement: Supplementary file 11 — Original Western blot Figures [file 41420_2025_2414_MOESM11_ESM.pdf]

Original Western blot Figures

Fig. 2B

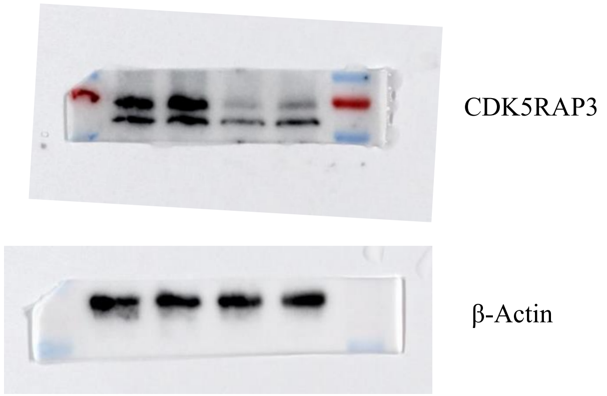

Fig. 4C

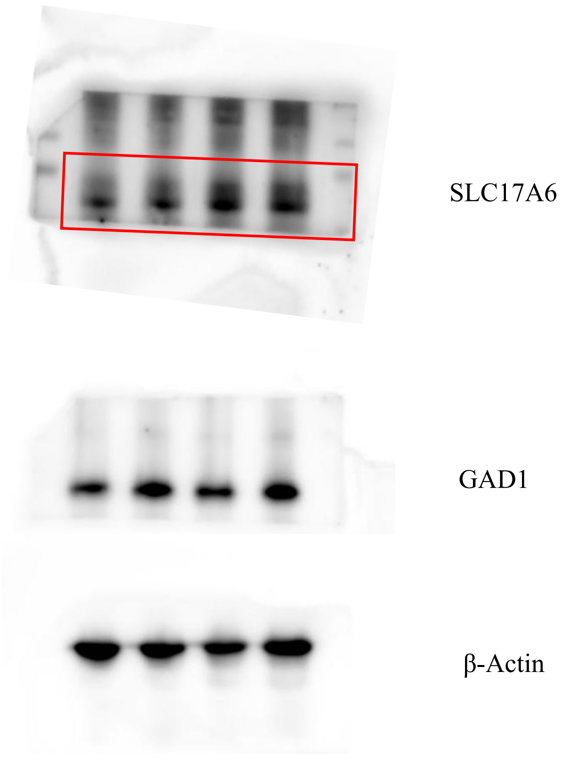

Fig. 5C

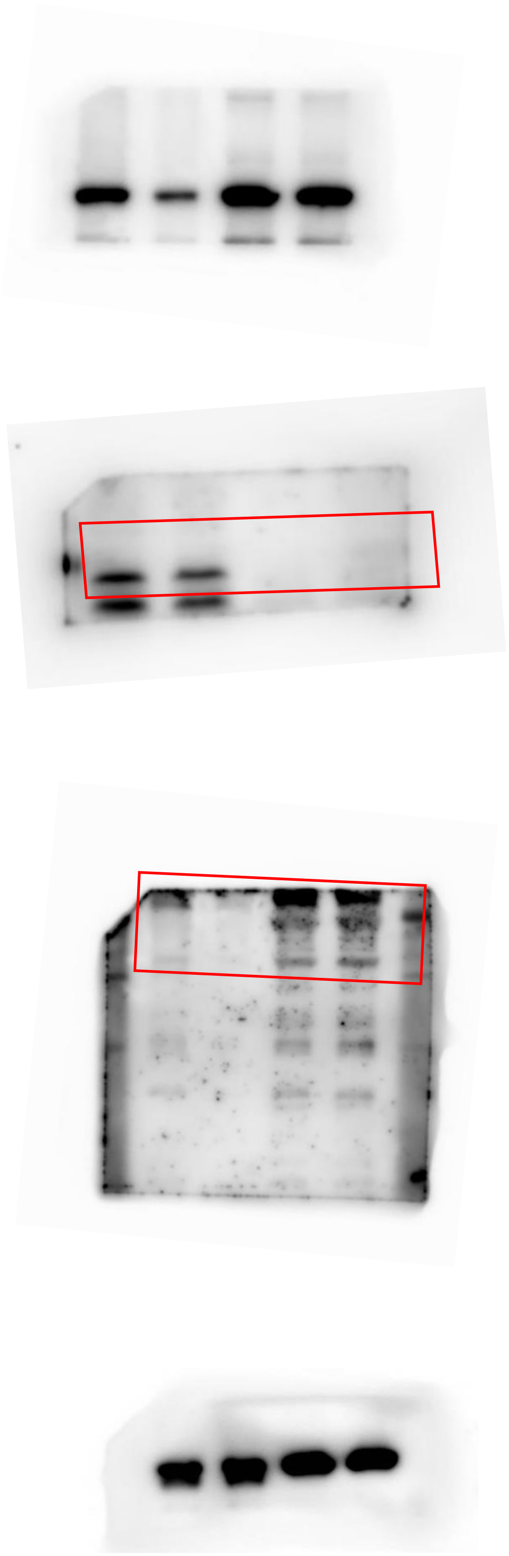

GRP78

CDK5RAP3

XBP1s

β-Actin

Fig. 5D

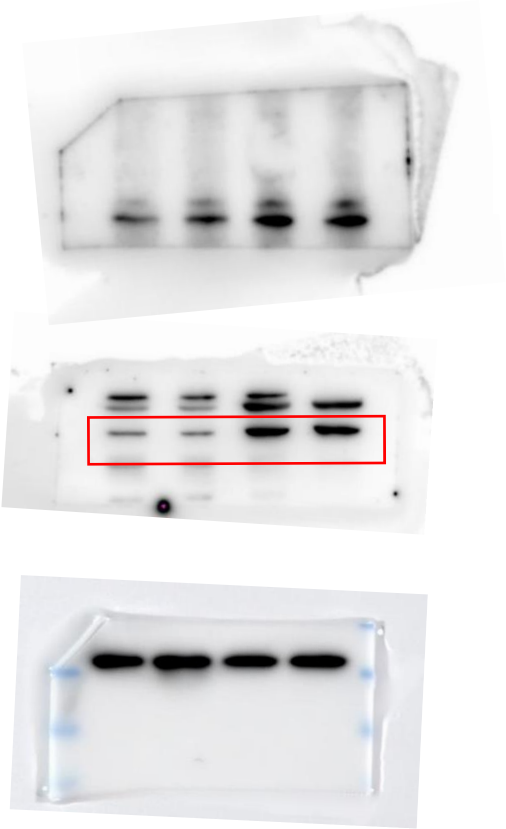

RPN1

ALG2

β-Actin

Fig. 5E

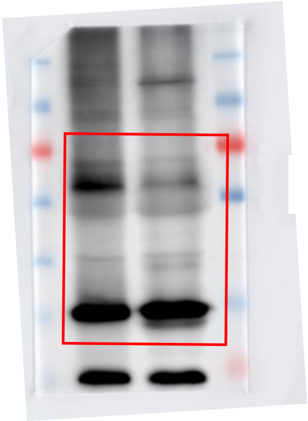

SLC17A6

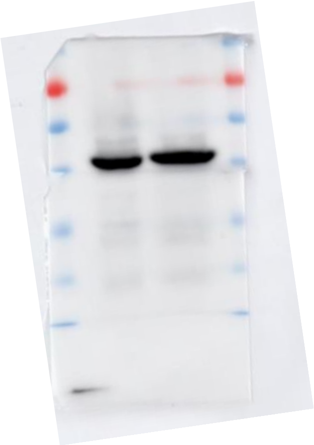

β-Actin

Fig. 6A

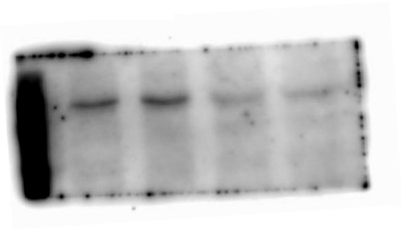

CDK5RAP3

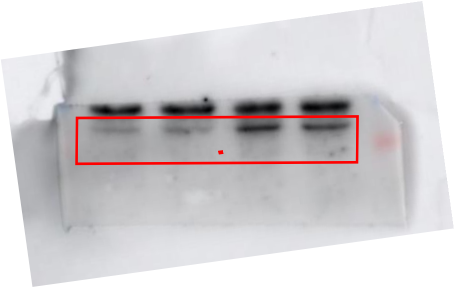

CHOP

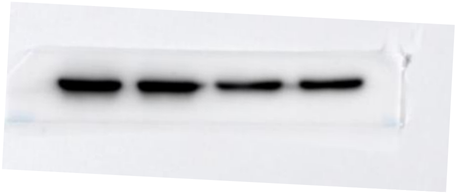

β-Actin

Fig. 6B

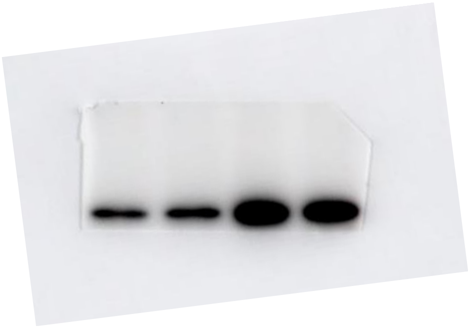

GRP78

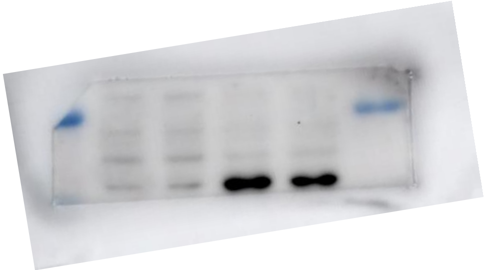

Caspase-12

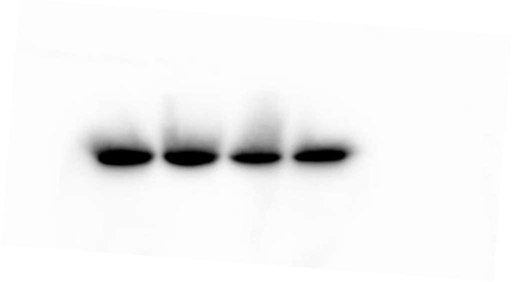

β-Actin

Fig. 6C

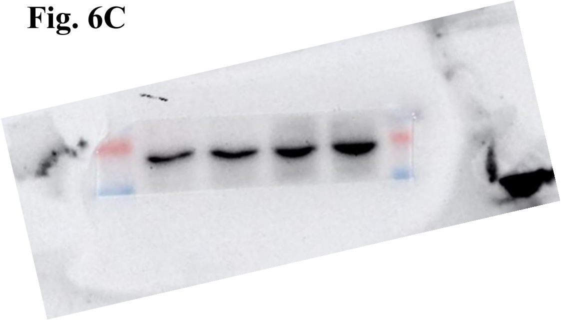

RPN1

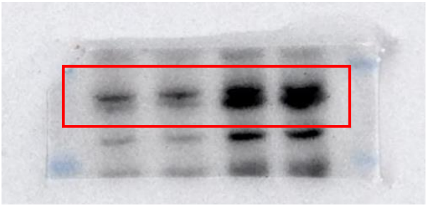

ALG2

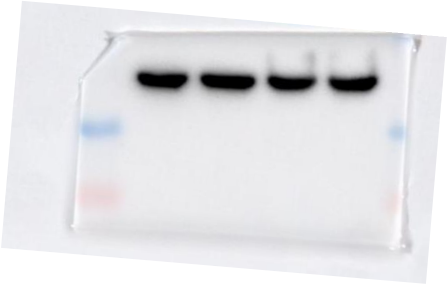

β-Actin

Fig. 7C

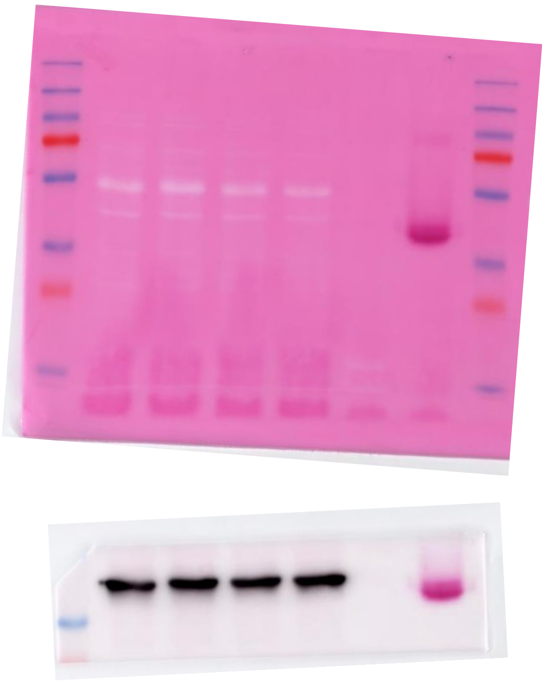

Non-Glycoprotein

β-Actin

Fig. 7D

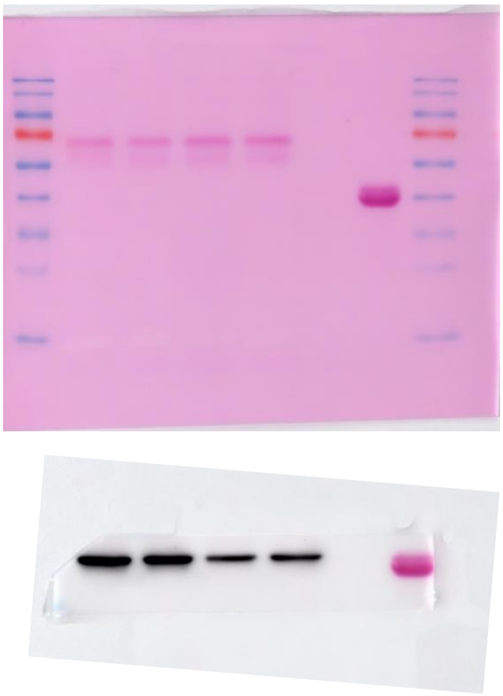

Glycoprotein

β-Actin

Fig. 8A

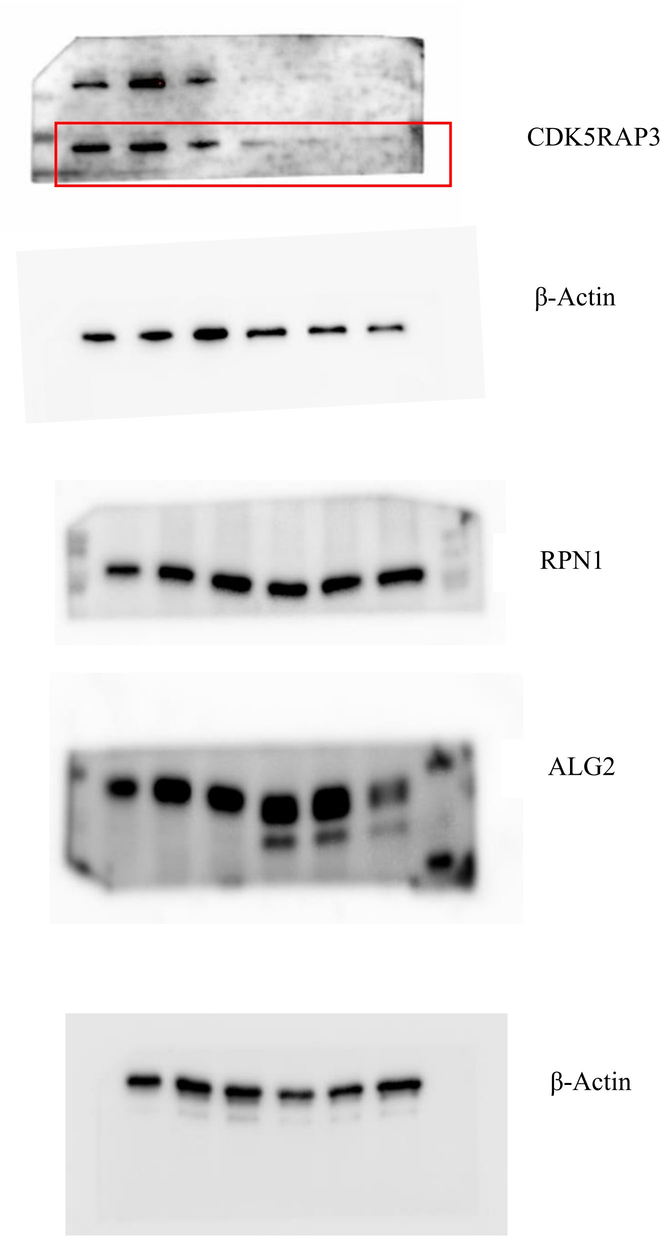

Fig. 8B

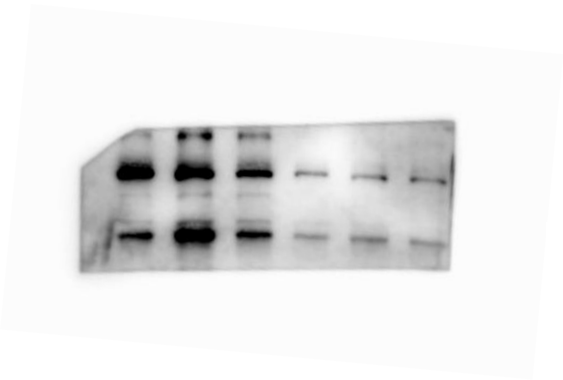

CDK5RAP3

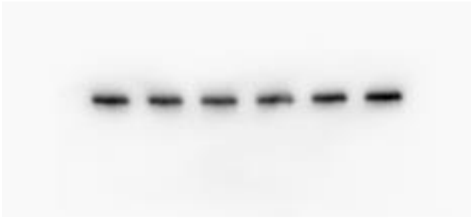

β-Actin

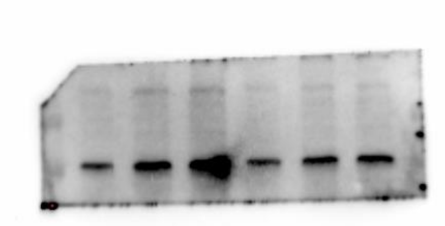

RPN1

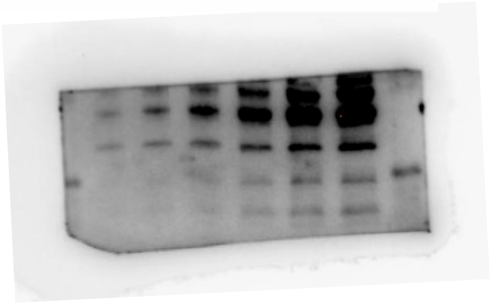

ALG2

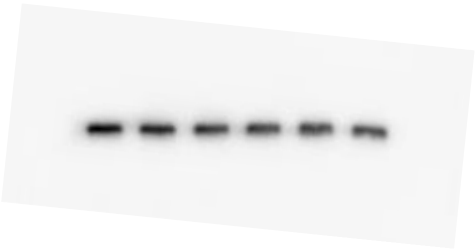

β-Actin

Fig. S3

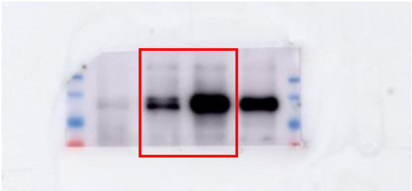

p-IRE1α

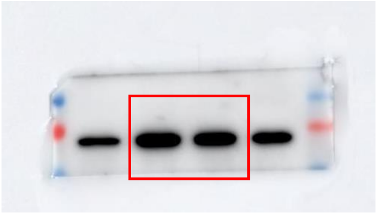

RPN1

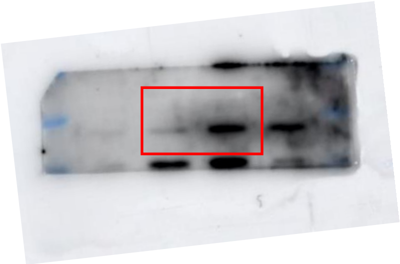

ALG2

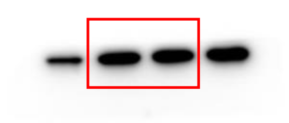

β-Actin

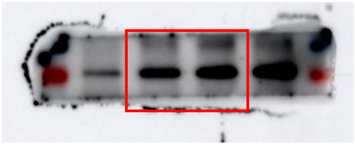

GRP78

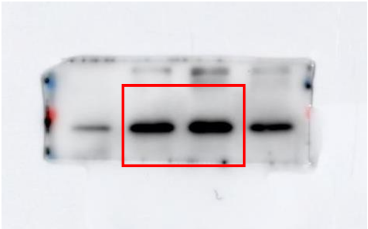

CDK5RAP3

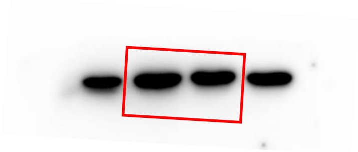

β-Actin

Fig. S6A

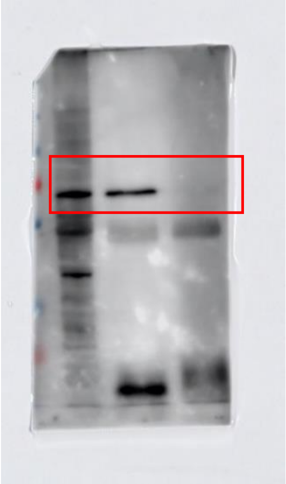

CDK5RAP3

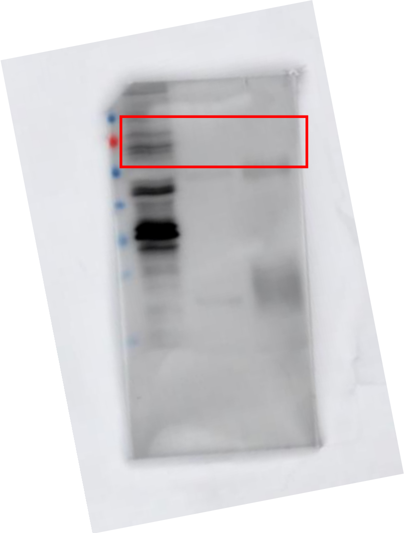

RPN1

Fig. S6B

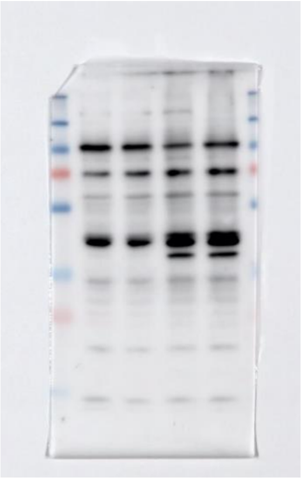

Ufm1

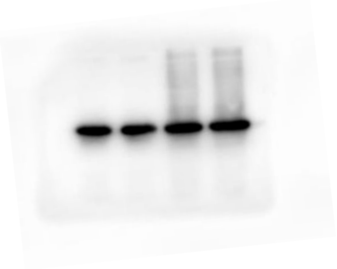

β-Actin

Fig. S6C

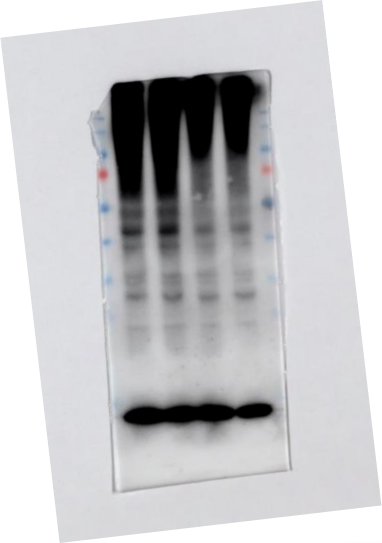

Ubiquitin

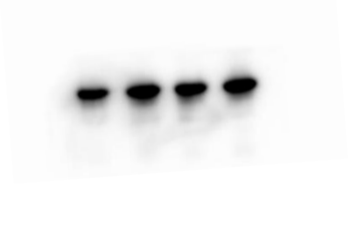

β-Actin
